# Erythrocyte‐Leveraged Oncolytic Virotherapy (ELeOVt): Oncolytic Virus Assembly on Erythrocyte Surface to Combat Pulmonary Metastasis and Alleviate Side Effects

**DOI:** 10.1002/advs.202303907

**Published:** 2023-11-23

**Authors:** Mingyang Liu, Ruizhe Zhang, Hanwei Huang, Pengfei Liu, Xu Zhao, Hu Wu, Ying He, Ruizhe Xu, Xifeng Qin, Zhenguo Cheng, Hongyu Liu, Onder Ergonul, Füsun Can, Defang Ouyang, Zhenning Wang, Zhiqing Pang, Funan Liu

**Affiliations:** ^1^ Department of Surgical Oncology and General Surgery The First Hospital of China Medical University 155 North Nanjing Street, Heping District Shenyang 110001 China; ^2^ Department of Pharmaceutics School of Pharmacy Fudan University and Key Laboratory of Smart Drug Delivery Ministry of Education Shanghai 201203 China; ^3^ Key Laboratory of Precision Diagnosis and Treatment of Gastrointestinal Tumors China Medical University Ministry of Education 155 North Nanjing Street, Heping District Shenyang 110001 China; ^4^ Sino‐British Research Centre for Molecular Oncology National Centre for International Research in Cell and Gene Therapy School of Basic Medical Sciences Academy of Medical Sciences Zhengzhou University Zhengzhou 450052 China; ^5^ Koç University Iş Bank Center for Infectious Diseases (KUISCID) Koç University School of Medicine and American Hospital Istanbul 34010 Turkey; ^6^ State Key Laboratory of Quality Research in Chinese Medicine Institute of Chinese Medical Sciences (ICMS) University of Macau Macau 999078 China; ^7^ Phase I Clinical Trials Center The First Hospital China Medical University 518 North Chuangxin Road, Baita Street, Hunnan District Shenyang Liaoning 110102 China

**Keywords:** biocompatibility, erythrocytes, hitchhiking, oncolytic virus, targeted delivery

## Abstract

Despite being a new promising tool for cancer therapy, intravenous delivery of oncolytic viruses (OVs) is greatly limited by poor tumor targeting, rapid clearance in the blood, severe organ toxicity, and cytokine release syndrome. Herein, a simple and efficient strategy of erythrocyte‐leveraged oncolytic virotherapy (ELeOVt) is reported, which for the first time assembled OVs on the surface of erythrocytes with up to near 100% efficiency and allowed targeted delivery of OVs to the lung after intravenous injection to achieve excellent treatment of pulmonary metastases while greatly improving the biocompatibility of OVs as a drug. Polyethyleneimine (PEI) as a bridge to assemble OVs on erythrocytes also played an important role in promoting the transfection of OVs. It is found that ELeOVt approach significantly prolonged the circulation time of OVs and increased the OVs distribution in the lung by more than tenfold, thereby significantly improving the treatment of lung metastases while reducing organ and systemic toxicity. Taken together, these findings suggest that the ELeOVt provides a biocompatible, efficient, and widely available approach to empower OVs to combat lung metastasis.

## Graphical abstract

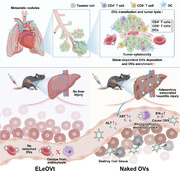

## 1 Introduction

Cancer has been a major threat to public health worldwide and one of the leading causes of death over the past few decades.^[^ ^1^ ^]^ Although the early detection, diagnosis, and treatment of cancer, the popular application of various cancer screening methods, as well as the close observation and follow‐up of precancerous lesions, have improved the survival rate of cancer patients to a certain extent, the current tumor treatment methods including surgery, chemotherapy, and radiation therapy are still not satisfactory if cancer is not detected and diagnosed early.^[^ ^1^, ^2^ ^]^ On the other hand, when most patients are diagnosed with cancer, the cancer often has formed distant metastases. Once it progresses to this point, the prognosis for patients is often poor.^[^ ^3^ ^]^ According to the International Agency for Research on Cancer (IARC), the most common metastatic site of various primary cancers is the lung due to high blood vessel density and abundant capillaries.^[^ ^4^ ^]^ In fact, almost all cancers have the potential to spread to the lungs, which can be fatal if left untreated. However, there is currently no efficient and specific treatment available for lung metastases.^[^ ^5^ ^]^

Oncolytic virus therapy (OVT) is a novel immunotherapy that induces antitumor responses through selective self‐replication within cancer cells and oncolytic virus (OV)‐mediated immune stimulation.^[^ ^6^ ^]^ In recent years, oncolytic virus immunotherapy has flourished in the field of tumor treatment.^[^ ^7^ ^]^ Four OV‐related products have been approved for marketing and more than 100 OVs drugs are in clinical trials.^[^ ^8^ ^]^ The anti‐tumor ability of OVs are mainly reflected in three aspects: directly and selectively infect and kill tumor cells; act on the stromal cells of the tumor microenvironment to generate neutrophil clumps in the blood vessels of the tumor, leading to the collapse of tumor blood vessel; activation of host innate and adaptive immune responses to tumors.^[^ ^6^, ^9^ ^]^ In addition, a large number of tumor antigens generated by cancer cell lysis are recognized and captured by antigen‐presenting cells (APCs), which then activate humoral and cellular immunity mediated by CD4^+^ and CD8^+^ T cells.^[^ ^10^ ^]^ Despite the incredible advantages of OVs in cancer therapy, OVs are not recommended in clinical treatment guidelines.^[^ ^11^ ^]^ The reason for this is that OV immunotherapy still suffers from an obvious deficiency: Currently, all OVs have to be administered intratumorally rather than intravenously due to severe organ toxicity, cytokine release syndrome (CRS), and extremely poor circulation.^[^ ^12^ ^]^ Moreover, for most lung metastases produced by tumors, it is particularly difficult for OVs to combat them by intratumoral injection due to the existence of multiple and deep micrometastases.^[^ ^13^ ^]^ Therefore, it is of great significance to develop a new strategy for OV treatment of lung metastases that is simple and feasible and can simultaneously improve the biocompatibility and therapeutic efficacy of OVs.^[^ ^11^, ^14^ ^]^

**Figure 1:**
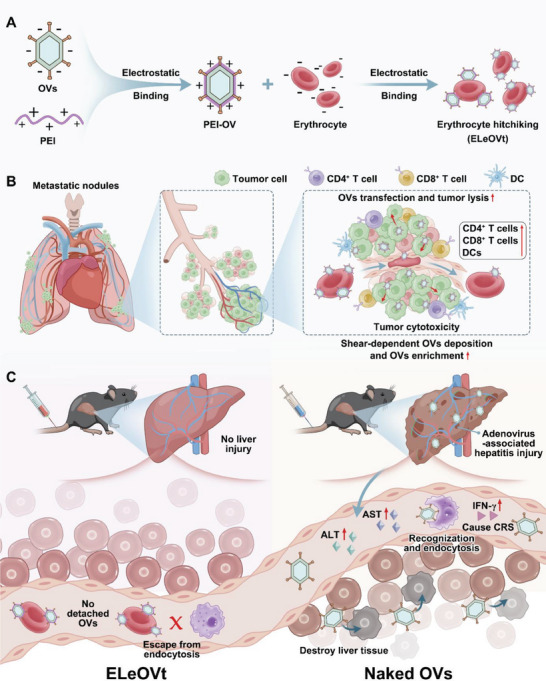
Schematic illustration of the preparation of ELeOVt and the mechanism of ELeOVt to improve the anti‐lung metastatic tumor effect and the biocompatibility of OVs. A) The construction of ELeOVt. B) The mechanism of ELeOVt to improve the anti‐lung metastatic tumor effect of OVs. C) The mechanism of ELeOVt to improve the biocompatibility of OVs.

To achieve efficient OV delivery to the lung enabling to effective treatment of lung metastases, we developed an erythrocyte‐leveraged oncolytic virotherapy (ELeOVt) strategy that assembled OVs on the surface of erythrocytes with high loading capability and efficiency using positive polyethyleneimine (PEI) to bridge negative OVs and erythrocytes through electrostatic interaction (**Figure** **1**A). ELeOVt could help OVs to escape the clearance of the mononuclear phagocyte system, extend OV circulation in the blood, and reduce systemic cytokine release after intravenous injection by leveraging the “don't eat me” signal of erythrocytes.^[^ ^15^ ^]^ In addition, ELeOVt had the potential to substantially reduce the amount of free OVs in the blood, thus preventing OVs from entering the hepatic parenchyma, destroying large amounts of liver tissue, as well as causing dramatic increases in ALT and AST levels. When experiencing high shear forces in stenotic pulmonary capillaries and vessels of tumor metastasis nodules, ELeOVt could responsively dislodge OVs that were not firmly bound on the erythrocyte surface, which could improve OV targeting to lung metastases. At the same time, PEI‐modification on detached OVs could increase the uptake of OVs by tumor cells and enhance the transfection effect of OVs in tumor cells, thus greatly improving the anti‐tumor efficacy of OVs and promoting the tumor immune microenvironment, including tumor lysis, tumor cytotoxicity and the increase in the amount of CD4^+^, CD8^+^ T cells, and DCs. Therefore, ELeOVt could not only reduce organ toxicity and systemic cytokine release syndrome of intravenous OVs but also enhance the therapeutic efficacy of OVs for lung metastases (Figure 1B,C). In this study, the loading capability and efficiency of OVs in ELeOVt, the shear force‐responsiveness, the cellular uptake and transfection behaviors, the circulation and biodistribution profiles, the targeting property to lung metastases, the toxicity, the tumor immune microenvironment, and the anti‐metastasis efficacy of ELeOVt were verified. In general, ELeOVt provides a biocompatible, efficient, and widely available approach to combat lung metastases.

## 2 Results

### 2.1 Generation and Characterization of ELeOVt

**Figure 2:**
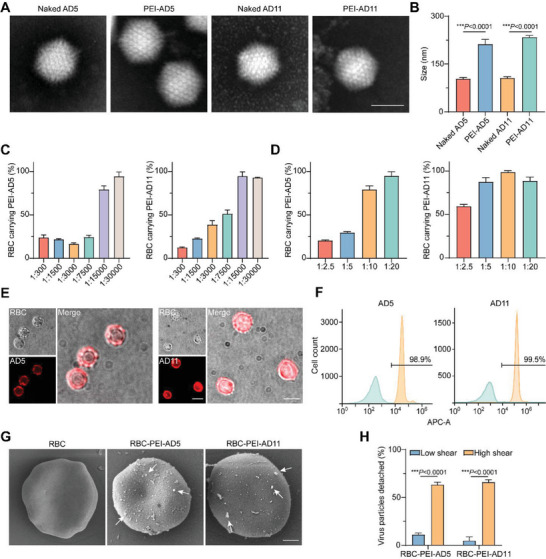
Preparation and characterization of ELeOVt. A) Transmission electron microscopy (TEM) images of naked AD5, PEI‐AD5, naked AD11, and PEI‐AD11, scale bar = 100 nm. B) The particle size of naked AD5, PEI‐AD5, naked AD11, and PEI‐AD11, *n* = 3. C) OV binding efficiency on mouse erythrocytes at different mole ratios of OVs to PEI, *n* = 3. D) OV binding efficiency on mouse erythrocytes at different mole ratios of erythrocytes to PEI‐AD, *n* = 3. E) CLSM images of ELeOVt, scale bar = 5 µm. F) Percentage of mouse erythrocytes carrying OVs. G) Scanning Electron Microscope (SEM) images of ELeOVt, AD was marked by the white arrow, scale bar = 1 µm. H) ELeOVt could specifically detach OVs under shear stress similar to that in lung vessels. Samples were sheared for 30 min (*n* = 3). Low shear indicated rotary shear at ≈1 Pa, while high shear was at 6 Pa. Data are displayed as mean ± s.d. The data in (C), (D), and (H) are presented as the percentage of the initial dose of OVs. Statistical significance was analyzed by a two‐tailed Student's t‐test. *p*‐value: ***p < 0.001.

We used adenovirus 5 (AD5) and adenovirus 11 (AD11) as model OVs in this study. OVs were added to the PEI solution to obtain PEI‐coated OV (PEI‐AD) and then assembled on mouse erythrocytes to generate RBC‐PEI‐AD (namely ELeOVt). Transmission electron microscopy (TEM) imaging and dynamic laser scattering demonstrated that PEI coating on OVs did not change the shape of OVs but significantly increased the diameter of OVs, indicating the formation of the cationic polymer coating (**Figure** **2**A,B). The results showed that the zeta potential of AD5 notably increased from −16.54 ± 1.11 to +21.75 ± 0.61 mV after PEI wrapping. Similarly, the zeta potential of AD11 notably increased from −14.82 ± 1.30 to +27.4 ± 0.78 mV after PEI wrapping (Figure S1, Supporting Information). Then we evaluated the loading capability and efficiency of OVs in RBC‐PEI‐AD (Figure 2C,D). It was found that the loading efficiency of OVs in RBC‐PEI‐AD reached > 80% when the mole ratio of OVs to PEI was 1:15 000 or lower, regardless of whether the OV was AD5 or AD11 (Figure 2C). Since PEI, as a cationic polymer, may cause OV agglomeration and erythrocyte damage at a very high concentration, we prepared RBC‐PEI‐AD by fixing the mole ratio of OVs to PEI at 1:15 000. It was also found that the loading efficiency of OVs in RBC‐PEI‐AD increased with the mole ratio of OVs to RBCs (Figure 2D). When incubating erythrocytes with PEI‐AD at a 1:10 mole ratio of RBCs to OVs, more than 80% of OVs were assembled on erythrocytes regardless of AD5 or AD11. The number of OVs bound to erythrocytes was also quantified. Due to the high binding efficiency of PEI‐AD to erythrocytes, mouse erythrocytes were able to carry high viral doses (as high as 1.75 × 10^8^ pfu or 17.5 × 10^9^ viral particles per 10^9^ erythrocytes) (Figure S2, Supporting Information). More importantly, the loading capability of OVs can be easily tuned by manipulating the feed mole ratio of OVs to erythrocytes. Confocal laser scanning microscopy (CLSM) images revealed that red AD‐Cy5.5 co‐localized well with the erythrocytes, which confirmed the efficient assembly of OVs on mouse erythrocytes (Figure 2E). Flow cytometry results displayed that up to 98% of erythrocytes carried Cy5.5‐labeled OVs (Figure 2F), further indicating the excellent loading efficiency of OVs in RBC‐PEI‐AD. Scanning electron microscopy (SEM) also demonstrated that OVs successfully assembled on mouse erythrocytes and this assembly did not change the biconcave disc‐like cell morphology of erythrocytes, indicating that little damage was caused to erythrocytes using the assembling method (Figure 2G). All these results suggest that RBC‐PEI‐AD has high loading capability and efficiency of OVs and can be finely tuned according to demand of OV dose. To explore the shear force‐responsiveness, ELeOVt was sheared for 30 min under low (≈1 Pa) or high (6 Pa) shear stress. As shown in Figure 2H, the separation of PEI‐coated AD from the surface of mouse red blood cells obviously depended on the shear force. Under the low shear force environment, only a small amount of OVs were separated from the surface of red blood cells. However, under the high shear force environment, most OVs fell off from the surface of red blood cells, providing a basis for the targeted delivery of OVs to the pulmonary metastatic tumor nodes.

### 2.2 Erythrocytes Reduced the Interaction of OVs with Macrophages

**Figure 3:**
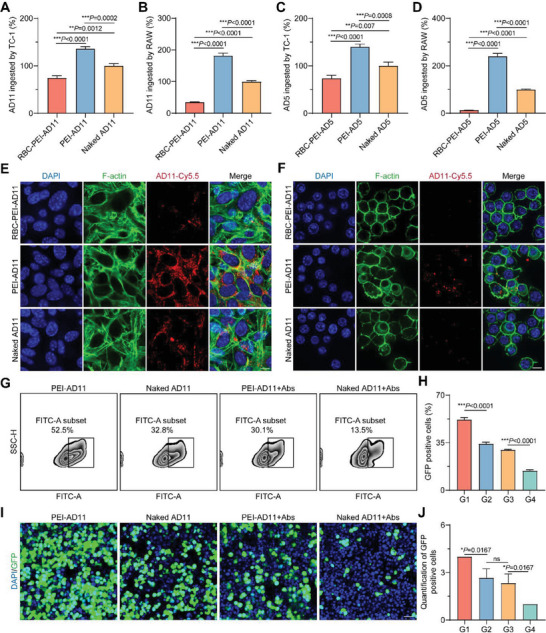
ELeOVt reduced the interaction of OVs with macrophages. A,B) The relative percentage of RBC‐PEI‐AD11, PEI‐AD11, and naked AD11 endocytosed by A) TC‐1 cells and (B) macrophages, *n* = 3. C,D) The relative percentage of RBC‐PEI‐AD5, PEI‐AD5, and naked AD5 endocytosed by C) TC‐1 cells and D) macrophages, *n* = 3. E,F) CLSM images of Cy5.5‐labeled RBC‐PEI‐AD11, PEI‐AD11, and naked AD11 endocytosed by E) TC‐1 cells and F) macrophages, scale bar = 10 µm. G,H) The transfection efficiency of PEI‐AD11 and naked AD11 on HCT 116 cells in the presence of anti‐ad11 neutralizing antibodies determined by G) flow cytometry, (H) the corresponding quantitative results, *n* = 3. I) CLSM images and J) quantitative score of HCT 116 cells transfected with different AD11 formulations, scale bar = 40 µm, *n* = 3. G1: PEI‐AD11, G2: naked AD11, G3: PEI‐AD11+Abs, G4: naked AD11+Abs. Data are displayed as mean ± s.d.. The data in (A–D) are presented as a relative percentage compared to the naked AD11 or AD5 group. Statistical significance was analyzed by one‐way ANOVA with a Tukey *post hoc* test; ns means no significant difference. *p*‐value: *p  <  0.05, **p  <  0.01, ***p  <  0.001.

Next, we investigated whether OVs assembled on erythrocytes could escape phagocytosis by mononuclear macrophages in the blood circulation in vivo through the “umbrella” effect provided by erythrocytes and whether ELeOVt could increase the uptake of AD by tumor cells at the lung metastasis site. We first verified it in an in vitro cellular assay. As shown in **Figure** **3**A,B, the proportion of AD11 uptake by cells was quantified by qPCR after co‐incubation of AD11 preparations (including RBC‐PEI‐AD11, PEI‐AD11, and naked AD11) with tumor cells or macrophages. It was found that the uptake of AD11 by TC‐1 and RAW 264.7 cells was significantly increased for the PEI‐AD11 group compared to the naked AD11 group, which was 1.36 and 1.81 times higher than that of the naked AD11 group, respectively. After assembling PEI‐AD11 on the erythrocyte surface, the uptake of AD11 by RAW 264.7 and TC‐1 cells was diminished, 0.35 and 0.74 times that of the naked AD11 group, respectively (Figure 3A,B). It suggests that erythrocytes can prevent other cells from easily tearing OVs off the erythrocyte surface and engulfing them in the cell. Similar results were shown for AD5 preparations (Figure 3C,D). In addition, the ability of RBC‐PEI‐AD11 or RBC‐PEI‐AD5 to resist macrophage endocytosis did not significantly change with the loading ratio of RBCs to OVs (Figure S3, Supporting Information). CLSM images of cellular uptake of AD11 or AD5 preparations also successfully confirmed the increased cellular uptake effect of PEI coatings on OVs and the protective effect of ELeOVt on OV uptake from cells (Figure 3E,F; Figures S4 and S5, Supporting Information).

Since the uptake of PEI‐coated OVs by tumor cells was significantly increased compared to OVs, we believed that this increased uptake was accompanied by a concomitant increase in the transfection rate and the ability of OVs to kill tumor cells. We used AD11, which has a green fluorescent protein (GFP) gene to examine the ability of PEI wrapping to enhance AD11 transfection into cells. As shown in Figure 3G, naked AD11 could transfect 34.1 ± 1.35% of HCT 116 cells to express GFP fluorescence, while PEI‐AD11 had the ability to transfect 51.93 ± 1.63% of cells. Another important reason for the current limited clinical use of OVs is that OVs require repeated injections to achieve sustained tumor suppression, and repeated injections can easily cause the body to develop specific neutralizing antibodies against OVs.^[^ ^13a^ ^]^ Therefore, we used AD11‐neutralizing antibodies pre‐incubated with PEI‐AD11 and naked AD11 to model whether the AD11 formulation could still exert its original transfection effect on tumor cells in the presence of neutralizing antibodies in vivo. Compared to the transfection efficiency of 14.3 ± 0.72% in the naked AD11 group in the presence of neutralizing antibodies, the PEI‐AD11 group maintained a transfection efficiency of 29.7 ± 0.35% after pre‐incubation with neutralizing antibodies, which was similar to that of the naked AD11 group without neutralizing antibodies (Figure 3G,H). The inhibition rates of the neutralizing antibody on the transfection efficiency of naked AD11 and PEI‐AD11 were 58.1% and 42.8%, respectively. These results suggest that the PEI wrapped on AD11 can increase the transfection efficiency of AD11 and resist the sealing effect of the neutralizing antibody on the surface ligand of AD11 to a certain extent. CLSM images of HCT 116 cells transfected with AD11 formulations agreed well with the flow cytometry results (Figure 3I,J). We then examined whether PEI coatings on OVs could enhance the killing ability of OVs for tumor cells. As shown in Figures S6 and S7 (Supporting Information), various concentrations of PEI alone that we used in OV formulations had almost no effect on cell viability, while the killing ability of PEI‐coated OV on TC‐1 and HCT 116 cells was enhanced compared with naked AD.

### 2.3 ELeOVt Extended OV Circulation and Enhanced Targeted Delivery of OVs to Lung Metastases

**Figure 4:**
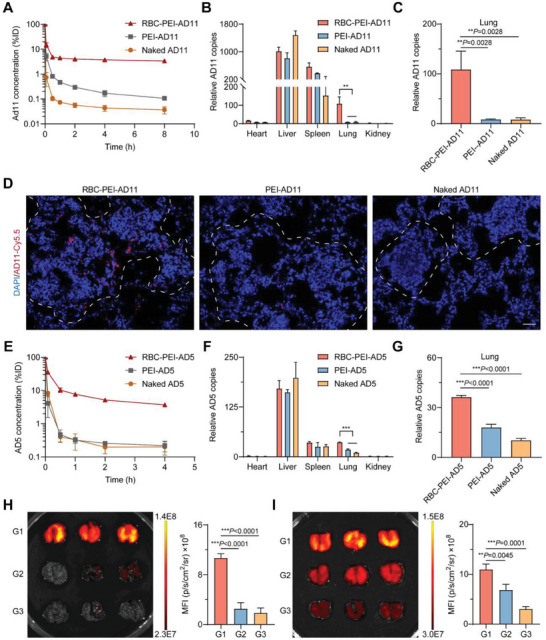
ELeOVt promoted circulation time and lung metastasis‐targeted enrichment of OVs. A) The circulation profiles of RBC‐PEI‐AD11, PEI‐AD11, and naked AD11 after intravenous administration, *n* = 3. B,C) The biodistribution of each AD11 formulation in B) vital organs and C) lungs with cancer metastases, *n* = 3. D) Cy5.5‐labeled AD11 distribution in the diseased lung at 4 h after intravenous administration of different AD11 formulations, the lung metastatic node was indicated within dotted lines, scale bar = 50 µm. E) The circulation profiles of RBC‐PEI‐AD5, PEI‐AD5, and naked AD5 after intravenous administration, *n* = 3. F,G) The biodistribution of each AD5 formulation in F) vital organs and G) lungs, *n* = 3. H) IVIS images and the corresponding fluorescence intensity of the diseased lungs at 4 h after intravenous injection of Cy5.5‐labeled RBC‐PEI‐AD11 (G1), PEI‐AD11 (G2), and naked AD11 (G3), *n* = 3. I) IVIS images and the corresponding fluorescence intensity of the lungs at 4 h after intravenous injection of Cy5.5‐labeled RBC‐PEI‐AD5 (G1), PEI‐AD5 (G2), and naked AD5 (G3), *n* = 3. Data are displayed as mean ± s.d.. The data in (A) and (E) are presented as the percentage of the injected dose (%ID). The data in (B), (C), (F), and (G) are presented as relative copies compared to the PBS group. Statistical significance was analyzed by one‐way ANOVA with a Tukey *post hoc* test. *p*‐value: **p  <  0.01, ***p  <  0.001.

We next performed pharmacokinetic and biodistribution studies of ELeOVt to examine the circulation profile as well as to test whether OVs could be shed in the pulmonary capillaries and deposited in the metastatic lungs due to in vivo pulmonary shear (**Figure** **4**). As shown in Figure 4A,E, for both OVs, AD11, and AD5, significantly higher OV concentrations in the blood were achieved in the RBC‐PEI‐AD group compared to the PEI‐AD and naked AD groups at all time points after intravenous injection. The concentration of AD11 in the blood at 8 h after injection was only 0.10%ID and 0.04%ID in the PEI‐AD11 and naked AD11 groups, respectively, but it was 3.36%ID in the RBC‐PEI‐AD11 group. In its entirety, ELeOVt increased the amount of AD11 circulating in the blood by >30‐fold. The concentration of AD5 present in the circulation at 4 h after injection was 0.22%ID and 0.20%ID in the PEI‐AD5 and naked AD5 groups, respectively, but it was 3.69%ID in the RBC‐PEI‐AD5 group. ELeOVt also increased the amount of AD5 circulating in the blood by >15‐fold. Pharmacokinetic parameters analysis also revealed that ELeOVt has a significantly longer circulation time than other groups (Figure S8, Supporting Information).

Encouraged by the much longer circulation time of ELeOVt, we conducted a biodistribution study of ELeOVt in the TC‐1‐hCD46‐Luc lung metastasis mouse model using qPCR. As shown in Figure 4B,C, ELeOVt deposited 13.2 times more AD11 in the lung than PEI‐AD11 and naked AD11 at 4 h after injection. Similarly, ELeOVt also significantly increased AD5 deposition in the lung compared to PEI‐AD5 and naked AD5 (Figure 4F,G). We investigated the distribution of OVs detached from erythrocytes in the lung where cancer metastases occurred using AD11 as a model OV. As shown in Figure 4D, consistent with the biodistribution results, more AD was found in lung sections of the RBC‐PEI‐AD11 group compared to the naked AD11 and PEI‐AD11 groups. More importantly, most, though not all, of the deposited AD11 penetrated deep into the tumor nodule of lung metastases, suggesting that OVs assembled on erythrocytes are able to precisely target and enrich at the metastasis site where they are needed to function. Next, we validated the enrichment of ELeOVt in the lung with cancer metastases via the In Vivo Imaging System (IVIS). It could be found that ELeOVt showed significantly stronger OV enrichment in the lung compared with PEI‐AD and naked AD (Figure 4H,I), which were well consistent with the biodistribution results determined by qPCR method (Figure 4C,G). All these results indicate that ELeOVt can achieve excellent long circulation and lung metastasis targeting.

### 2.4 ELeOVt Reduced Organ Toxicity and CRS of OVs

**Figure 5:**
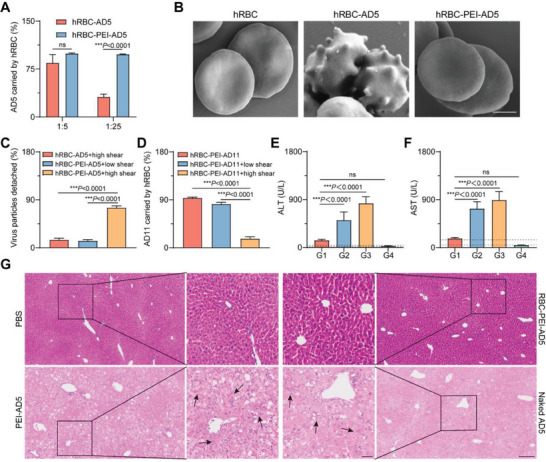
ELeOVt reduced toxicity of intravenous OVs. A) AD5 binding efficiency on human erythrocytes at different mole ratios of human erythrocytes to AD5, *n* = 3. B) The scanning electron microscope (SEM) images of human erythrocytes with AD5 assembled on them, scale bar = 5 µm. C) AD5 detached from human erythrocytes under different shear conditions, *n* = 3. D) The percentage of PEI‐AD11 binding to human erythrocytes under different conditions, *n* = 3. E) ALT and F) AST levels in the blood of mice at 72 h after intravenous administration of a high‐dose of AD5 formulations, the normal range for ALT or AST was located between the two dotted lines, *n* = 5. G1: RBC‐PEI‐AD5; G2: PEI‐AD5; G3: naked AD5; G4: PBS. G) The histopathological analysis of livers by H&E staining at 72 h after intravenous administration of a high‐dose of AD5 formulations, necrotic hepatocyte was marked by the black arrow, scale bar = 50 µm (inner) and scale bar = 200 µm (outer). Data are displayed as mean ± s.d.. The data in (A), (C), and (D) are presented as the percentage of the initial dose of OVs. Statistical significance was analyzed by a two‐tailed Student's t‐test in (A) and statistical significance in other data was analyzed by one‐way ANOVA with a Tukey post hoc test; ns means no significant difference. *p*‐value: ***p  <  0.001. The normal range of ALT and AST: ALT (13–43 U L^−1^), AST (59–156 U L^−1^).^[^ ^21^ ^]^

Severe organ and CRS greatly limit the clinical application of current OVs, and erythrocytes, an important component of self‐cells, are expected to overcome the poor biosafety of OVs.^[^ ^11^, ^16^ ^]^ According to some literature, AD5 may interact with human (but not murine) erythrocytes through the surface proteins (maybe coxsackie adenovirus receptor) and form bindings that can affect the systemic delivery of AD5. However, the direct binding of AD5 to human erythrocytes can also have adverse effects on these cells.^[^ ^17^ ^]^ We first verified whether AD5 would bind to human erythrocytes (hRBC) by co‐incubating AD5 and hRBC at a certain ratio. It was found that most of the naked AD5 or PEI‐AD5 were bound to hRBC at a 1:5 mole ratio of erythrocytes to AD5. However, as the amount of naked AD5 increased, the binding efficiency of AD5 to hRBC (hRBC‐AD5) started to decrease to only 31.3% at the 1:50 ratio. In contrast, the binding efficiency of PEI‐AD5 to hRBC (hRBC‐PEI‐AD5) was still maintained at 97.9% at the 1:50 ratio (**Figure** **5**A). This demonstrates that PEI‐AD5 binds to human erythrocytes in a manner that is not limited by the number of proteins on the erythrocyte surface, thus allowing for high OV loading on demand. We next took SEM images of hRBC, hRBC‐AD5, and hRBC‐PEI‐AD5, respectively (Figure 5B). Interestingly, the way AD5 binds to human erythrocytes by binding to the protein on the surface of erythrocytes caused significant morphological changes in most erythrocytes, which may cause damage to the erythrocytes. In contrast, after PEI wrapped AD5 and then bound to hRBC via electrical interaction, the erythrocytes still maintained a well‐behaved biconcave disc‐like cell morphology. We then investigated the release profiles of AD5 from hRBC‐AD5 and hRBC‐PEI‐AD5 under certain shear forces (Figure 5C). Similar to RBC‐PEI‐AD5, hRBC‐PEI‐AD5 showed only a small release at low shear and a large release at high shear. Due to the tight binding of AD5 to proteins, hRBC‐AD5 remained difficult to release under high shear conditions. We also verified the efficiency of PEI‐AD11 binding to hRBC and OV release at high and low shear, which remained consistent with the results of PEI‐AD5 (Figure 5D). As an exogenous and highly immunogenic biological particle, a large dose of OVs produces significant CRS‐associated side effects when injected intravenously, such as fever (89.5%), vomiting (57.9%), lymphocytopenia (47.4%), and leukocytopenia (31.6%).^[^ ^18^ ^]^ Therefore, we explored the in vivo safety of a single high‐dose injection of ELeOVt. As shown in Figures S9 and S10 (Supporting Information), naked AD and PEI‐AD caused an increase in IFN‐*γ* levels in the blood of mice compared with RBC‐PEI‐AD, suggesting a low antiviral immune response occurs in the RBC‐PEI‐AD group. Among the AD11 preparations, IL‐6 levels in the blood of mice in the naked AD11 and PEI‐AD11 groups were also significantly elevated compared to that in the RBC‐PEI‐AD11 group. The above results suggest that intravenous ELeOVt can partly avoid the inflammatory factor storms caused by the high immunogenicity of OVs and improve the biosafety of OVs in vivo. In clinical reports, adenovirus‐associated hepatitis is commonly seen in young children with incompletely developed immune systems and poor immunity, which has been reported in adults.^[^ ^19^ ^]^ Hepatotoxicity remains an unresolved problem for oncolytic adenovirus (OA) cancer therapy.^[^ ^20^ ^]^ We next checked whether a single injection of large doses of OVs would cause similar liver damage in C57 mice using AD5 as a model OV. As shown in Figure 5E–G, both naked AD5, and PEI‐AD5, upon entering the circulation, caused varying degrees of pathological damage to the liver due to the AD5 accumulation of the vast majority in the liver. They significantly elevated blood levels of glutamate transaminase (ALT) and glutathione transaminase (AST), which were more than tenfold those in the PBS group, showing significant abnormal liver functions (Figure 5E,F). In addition, they caused significant pathological damage to the liver, with necrosis and edema degeneration of hepatocytes, and fibrotic formation in the liver (Figure 5G). Although ALT and AST levels in the RBC‐PEI‐AD5 group were still elevated compared to the PBS group, they were substantially lower than those in the naked AD5 and PEI‐AD5 groups, suggesting ELeOVt could drastically reduce AD5‐caused liver damage. H&E sections of the livers of mice in both RBC‐PEI‐AD5 and PBS groups also showed that the liver was largely structurally intact with good cell morphology, and no obvious pathological damage was observed. Moreover, no noteworthy pathologic damage was observed in other major organs including the heart, spleen, lung, and kidneys of each group (Figure S11, Supporting Information). All these results indicate that ELeOVt can significantly avoid OV‐associated organ damage and CRS and improve the safety of OVs for tumor treatment.

### 2.5 ELeOVt Significantly Inhibited Lung Metastasis Progression

**Figure 6:**
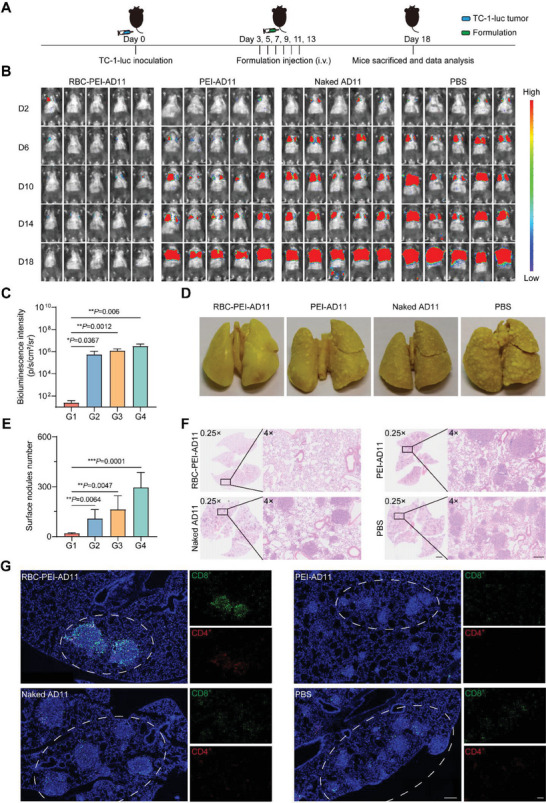
ELeOVt promoted the anti‐metastatic tumor efficacy of AD11. A) Schematic illustration of dosing regiments. B) IVIS bioluminescence images of model mice after different treatments, *n* = 5. C) Bioluminescence intensity of lung metastases at day 18, G1: RBC‐PEI‐AD11; G2: PEI‐AD11; G3: naked AD11; G4: PBS, *n* = 5. D) Representative lung photographs in different groups. E) The number of metastatic nodules in lungs after different treatments, G1: RBC‐PEI‐AD11; G2: PEI‐AD11; G3: naked AD11; G4: PBS, *n* = 5. F) H&E staining of lung tissues post‐treatment, scale bar = 2 mm (0.25×) and scale bar = 200 µm (4×). G) Representative immunofluorescence images of CD8^+^ (Green) and CD4^+^ (Red) T cells in lung tissues after different treatments, scale bar = 200 µm. Data are displayed as mean ± s.d.. Statistical significance was analyzed by one‐way ANOVA with a Tukey post hoc test. *p*‐value: *p  <  0.05, **p  <  0.01, ***p  <  0.001.

To evaluate the antitumor efficacy of ELeOVt, we constructed a mouse lung metastatic tumor model by injecting bioengineered TC‐1‐hCD46‐Luc cells into mice from the tail vein, and the treatment protocol was shown in **Figure** **6**A. Anti‐tumor efficacy of RBC‐PEI‐AD11, PEI‐AD11, Naked AD11, and PBS was determined by bioluminescence imaging with the In Vivo Imaging System (PerkinElmer, USA). As shown in Figure 6B and Figure S12 (Supporting Information), the bioluminescence imaging results showed that lung metastasis progression was significantly inhibited after RBC‐PEI‐AD11 treatment, while the other treatment modalities had little effect on lung metastasis progression. The individual bioluminescence intensity‐time curves for each mouse were shown in Figure S13 (Supporting Information), demonstrating the superior anti‐pulmonary metastasis efficacy of RBC‐PEI‐AD11 compared to other treatments. The bioluminescence intensity in the lungs of the RBC‐PEI‐AD11 group largely disappeared at day 18 after tumor cell inoculation compared with that of the PEI‐AD11, Naked AD11, and PBS groups (Figure 6C). Pathological sections and *ex vivo* lung photographs also clearly showed that the lungs of mice in the PEI‐AD11, Naked AD11, and PBS groups were occupied by pulmonary metastases, whereas almost no pulmonary metastases were observed in the RBC‐PEI‐AD11 group (Figure 6D,F; Figure S14, Supporting Information). The number of pulmonary nodules in the RBC‐PEI‐AD11 group was significantly lower than that in the other groups, which was only 6.6%, 12.0%, and 18.1% of that in the PBS, naked AD11, and PEI‐ AD11 groups, respectively (Figure 6E). There were no significant differences in the body weight of mice in all groups during the treatment (Figure S15, Supporting Information). We next evaluated the immune response at the tumor nodes and found that the RBC‐PEI‐AD11 group had abundant infiltration of CD8^+^ and CD4^+^ T cells at the tumor nodes, while the remaining three groups had only a small amount of T cell infiltration (Figure 6G; Figure S16, Supporting Information). In addition, higher expressions of IL‐6, TNF‐*α*, and IFN‐*γ* were expressed in lung metastatic nodes of the RBC‐PEI‐AD11 group compared with other groups to promote anti‐tumor immune response (Figure S17, Supporting Information). More apoptotic cells were observed in lung metastatic nodes of mice in the RBC‐PEI‐AD11 group compared with the PEI‐AD11, naked AD11, and PBS groups (Figure S18, Supporting Information). Finally, we evaluated the toxicity of ELeOVt at therapeutic doses to major organs in vivo (heart, liver, spleen, lung, and kidney) by H&E staining for histopathological analysis (Figure 6F; Figure S19, Supporting Information). No significant pathological changes and structural damage were observed in all major organs. The above results strongly suggest the superior anti‐pulmonary metastasis effect of biocompatible RBC‐PEI‐AD11.

**Figure 7:**
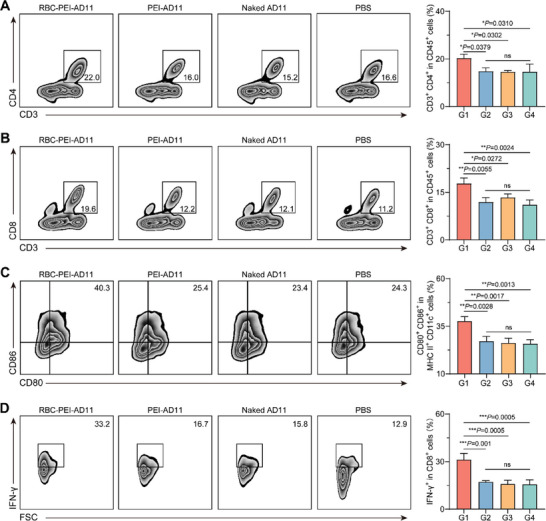
ELeOVt promoted the anti‐metastatic tumor immune response. A) Representative flow cytometry plots and the corresponding quantitative analysis of CD3^+^CD4^+^ T cells gating on CD45^+^ cells in lungs, *n* = 3. B) Representative flow cytometry plots and the corresponding quantitative analysis of CD3^+^CD8^+^ T cells gating on CD45^+^ cells in lungs, *n* = 3. C) Representative flow cytometry plots and the corresponding quantitative analysis of CD80^+^CD86^+^ cells gating on MHC II^+^CD11c^+^ cells in lungs, *n* = 3. D) Representative flow cytometry plots and the corresponding quantitative analysis of IFN‐*γ* ^+^ cells gating on CD8^+^ cells in lungs, *n* = 3. G1: RBC‐PEI‐AD11; G2: PEI‐AD11; G3: naked AD11; G4: PBS. Data are displayed as mean ± s.d.. The C57BL/6J mice were used as an animal model for metastatic lung cancer, and the sampling time for analysis was set to be the day following the third injection. Statistical significance was analyzed by one‐way ANOVA with a Tukey post hoc test; ns means no significant difference. *p*‐value: *p  <  0.05, **p  <  0.01, ***p  <  0.001.

The infection and replication of OVs in the tumor ultimately lead to cancer cell lysis, releasing large amounts of tumor antigens and inflammatory mediators, which promote an anti‐tumor immune response. Encouraged by the increased CD4^+^ and CD8^+^ T cell infiltration and up‐regulated expression levels of inflammatory factors in lung metastatic tumor nodules as shown by sectioning results, we investigated the anti‐tumor immune responses induced by ELeOVt. As shown in **Figure** **7**A,B and Figure S20 (Supporting Information), the amounts of CD4^+^ T cells and CD8^+^ T cells in the lungs of the RBC‐PEI‐AD11 group were significantly higher than those in other groups. Notably, the amount of CD80^+^CD86^+^ DCs in the lungs of the RBC‐PEI‐AD11 group was 1.39, 1.44, and 1.46 times higher than that in the PEI‐AD11, naked AD11, and PBS groups, respectively (Figure 7C; Figure S21, Supporting Information). Furthermore, the amount of IFN‐*γ* ^+^CD8^+^ T cells in the RBC‐PEI‐AD11 group was also significantly higher than that in other groups (Figure 7D; Figure S22, Supporting Information). Taken together, these results suggested that ELeOVt could promote the tumor immune microenvironment.

## 3 Discussion

In this study, we reported an efficient method for Erythrocyte‐leveraged oncolytic virotherapy (ELeOVt): the surface electrical properties of OVs were altered by PEI‐coating of OVs so that they were almost completely assembled on the erythrocyte surface, with a holdout efficiency of more than 90%. Due to the upper limit of the number of deliverable erythrocytes in both clinical patient treatment and animal experiments, only high‐efficiency and high‐dose OVs held on top of a single erythrocyte can achieve the desired dose for tumor treatment. Our data showed that model OVs (in this case AD5 and AD11) could be assembled on mouse and human erythrocytes with very high binding efficiency through the ELeOVt approach and were fully capable of meeting the dosing requirements. Also, our experimental data showed that the total host dose could be easily adjusted by varying the mole ratios of OVs to PEI and erythrocytes to PEI‐AD, thus providing the possibility to adjust the dose of OVs according to the specific lung metastases or disease stages. Primary tumors with lung metastases come from a wide range of sources, such as breast cancer, gastric cancer, intestinal cancer, and melanoma.^[^ ^22^ ^]^ The ELeOVt approach promises to be a versatile platform for the delivery of biotherapeutic OVs by loading different lung metastases with the corresponding OV product, depending on the primary tumor. The impact of different OVs on the performance of the ELeOVt method will be further explored in future studies. This approach has considerable translational potential due to its simplicity and high assembly efficiency, but we need to explore it further.

Dendritic polymers have great potential for a variety of biomedical applications, including drug delivery, gene therapy, tissue engineering, immunoassays, and bioimaging.^[^ ^23^ ^]^ As a cationic polymer, PEI is widely used as a drug delivery vehicle due to its long‐chain polymeric structure and the ease of electrical modification on the nanoparticle's surface.^[^ ^24^ ^]^ For example, in previous studies on mRNA delivery, cationic polymer PEI has been shown to improve the delivery efficiency of mRNA and thus has been used as an efficient delivery vehicle for mRNA.^[^ ^25^ ^]^ Similarly, in the present study, as an intermediate bridge, PEI could not only change the surface electrical properties of OVs to facilitate the successful assembly of OVs on the surface of red blood cells, but also enhance the uptake of OVs by tumor cells, and further promote the transfection and killing effect of OVs on tumor cells afterward. Particularly, PEI coatings significantly increased the transfection efficiency of OVs in the presence of OV‐associated neutralizing antibodies, demonstrating the multiple functions of PEI in the ELeOVt approach.

Due to unique physiological characteristics, such as high vascular density and abundant capillaries, the lung is one of the most common sites of metastasis for various primary cancers. OVs, novel immunotherapy, induce anti‐tumor responses through selective self‐replication within cancer cells and OV‐mediated immune stimulation. In contrast, the lung is particularly well‐suited for OV immunotherapy due to its proximity to the central immune organ, thymus, and the presence of a large number of lymph nodes that are susceptible to immune cell infiltration.^[^ ^26^ ^]^ However, due to the severe CRS and serious organ toxicity associated with intravenous administration of OVs, the current clinical administration of OVs is local intratumoral injection rather than intravenous injection. On the other hand, distant metastases, such as lung metastases, often produce multiple tumor nodules, and some micro‐metastases are not undetectable by conventional imaging at the early stage, which further limits the effectiveness of OVs in treating metastases.^[^ ^11^, ^12^, ^20^ ^]^ Previous studies have used erythrocytes as drug carriers by assembling nanoparticles and drugs directly on the surface of erythrocytes, which could improve the targeting effect of nanoparticles and drugs to the lung and lung metastases despite the low assembly efficiency, thus achieving enhanced antitumor efficacy against melanoma lung metastases.^[^ ^27^ ^]^ Inspired by the strategy of erythrocyte‐hitchhiking for targeted delivery of small‐molecule drugs, we considered that OVs could also be assembled on the surface of erythrocytes by the bridge of PEI to improve their targeting effect on the lung and lung metastases while reducing the severe organ toxicity and CRS caused by off‐target OVs, which was different from virus‐loaded mammalian cells (such as macrophage or dying tumor cells) reported before.^[^ ^28^ ^]^ Erythrocytes, as OVs carriers, played an important role in the composition of the organism itself, providing a “don't eat me” signal through the CD47 protein, thus allowing OVs to avoid immune recognition by the mononuclear phagocyte system during circulation in the body and effectively prolonging the circulation of OVs in the blood. Our in vitro macrophage uptake assays and in vivo pharmacokinetic assays in mice successfully demonstrated the ability of ELeOVt to perform these functions. Also, in vivo distribution experiments demonstrated that ELeOVt could significantly increase the accumulation of OVs in the lung, both of which features contributed to a significant improvement in the treatment of lung metastases. In addition, in vitro data simulating the shear environment in the lung illustrated that the separation of the majority of OVs from the erythrocyte surface was shear‐dependent, which was the basis for the precise delivery of OVs to the lung metastasis site while reducing the organ toxicity and CRS by the ELeOVt method. Specifically, ELeOVt could deliver up to ten‐fold more OVs to the lungs compared to the other OV formulations. With the ELeOVt approach, we could achieve effective and rapid targeting of OVs to lung metastases, avoiding adverse effects on human erythrocytes, severe organ toxicity, and cytokine release syndrome due to the off‐target effect of OVs. Our in vivo efficacy data showed that ELeOVt could provide superior efficacy in lung metastases. In the treatment of lung metastasis models, ELeOVt could promote the tumor immune microenvironment and effectively trigger a strong T‐cell‐mediated anti‐tumor immune response, almost cured the lung metastases in mice. This two‐birds‐with‐one‐stone approach significantly reduces the toxicity of OVs and allows for better lung metastasis treatment while improving their biosafety.

## 4 Conclusion

In summary, we reported an efficient erythrocyte‐leveraged oncolytic virotherapy (ELeOVt) approach, which for the first time assembled OVs on the surface of erythrocytes with high efficiency and loading capability and precisely delivered OVs to the lung for the treatment of lung metastases using the physiological effect of high shear force present in the lung. By wrapping OVs with PEI as an intermediate bridge, the uptake, transfection, and killing effect of OVs on tumor cells could be significantly enhanced. The circulation time of OVs in the blood system could be effectively prolonged by the ELeOVt approach, increasing the targeting effect of OVs to the diseased lungs by an order of magnitude, thus significantly enhancing the effectiveness of OVs in treating lung metastases and promoting the tumor immune microenvironment. At the same time, the assembly of OVs on the surface of red blood cells could significantly improve their biosafety and reduce the risk of organ toxicity and cytokine release syndrome. In conclusion, the ELeOVt approach serves as a promising strategy not only for OVs to treat lung metastasis but also for other biological therapies against lung metastasis originating from different primary tumors, with strong translational potential.

## 5 Experimental Section

### Materials

The AD11‐tel (AD11) and AD5‐nsIL‐12 (AD5) were donated by Beijing Bio‐targeting Therapeutics Technology Co., Ltd (Beijing, China). The TC‐1‐hCD46 and TC‐1‐hCD46 with fluorescein (TC‐1‐hCD46‐Luc) were donated by Zhengzhou University, China. HCT 116 and RAW264.7 cells were obtained from the American Type Culture Collection (ATCC, USA). HCT 116 and RAW 264.7 were maintained in Dulbecco's modified Egle's medium (DMEM) containing 10% FBS. TC‐1‐hCD46‐Luc was maintained in DMEM containing 10% FBS (CLARK, USA), 1% blasticidin, and 1% puromycin. Poly(ethylenimine) (PEI, MW: 10 000 Da) was purchased from MACKLIN (Cat: E808880). C57BL/6J (C57) mice were purchased from Shanghai SLAC Laboratory Animal Co., Ltd. (Shanghai, China). All enzyme‐linked immunosorbent assay kits were purchased from Multi‐Sciences (China). The antibody information is listed in Table S1 (Supporting Information) in the Supporting Information. Animal protocols in this study were approved by the Animal Ethics Committee of China Medical University (CMU2022481). This study involved human erythrocyte samples and was approved by the Ethics Committee at The First Hospital of China Medical University (AF‐SOP‐07‐1,1‐01). All participating subjects gave an informed written consent form before participation in the study.

### Virus Titers Assay

The titers of AD5 and AD11 were as previously described with little modification.^[^ ^29^ ^]^ Briefly, JH‐293 cells were first seeded into 96‐well plates at 3000 cells per well and cultured overnight. OVs were added and incubated for 3 d. Finally, the titer of OVs was determined by GFP fluorescence instead of cytopathic effect (CPE).

### Preparation of EleOVt and Characterization

A certain mass of PEI was weighed into a 1.5 ml EP tube, and PBS was added to dissolve it uniformly. PEI‐AD was obtained by pipetting OVs into the PEI solution slowly and uniformly with different ratios of AD11 or AD5 particles to PEI molecules. The PEI‐AD was then resuspended in deionized water and assessed for their size, zeta potential using dynamic light scattering (ZETASIZER Pro, Malvern). The obtained PEI‐AD was then mixed with different ratios of erythrocytes to OV particles in an EP tube, and the tube was placed on a longitudinal spindle and rotary co‐incubation for 50 min. The hitchhiked erythrocytes (RBC‐PEI‐AD, EleOVt) were then pelleted by centrifugation at 500 g for 5 min at 4 °C, unabsorbed OV particles were carefully removed, and the pellet was washed again with 1 × PBS to remove loosely bound OV particles. The hitchhiked erythrocytes were finally resuspended at 10% (v/v) in 1 × PBS and used for further characterization or in vivo studies. AD11 and AD5 were labeled by NHS‐Cy5.5, and the co‐localization of OVs and erythrocytes was observed by Confocal laser scanning microscopy (CLSM, Olympus IXplore SpinSR) and detected by flow cytometry (CytoFlex S, Beckman Coulter). OVs assembly to erythrocytes were confirmed using SEM (Zeiss FESEM Supra 55VP, Zeiss FESEM Ultra 55). Briefly, the hitchhiked erythrocytes were fixed using a 2.5% glutaraldehyde solution and washed in an increasing ethanol gradient before being chemically dried using hexamethyldisilazane. Last, the samples were sputter coated (EMT 150T ES metal sputter coater, PA, USA) prior to imaging.

### Quantitative Real‐Time PCR

Quantitative PCR (qPCR) was performed as described previously.^[^ ^30^ ^]^ Briefly, DNA in cells or fresh tissues were extracted by using a Fast‐pure Cell/Tissue DNA Isolation Mini Kit (Vazyme, China). The qPCR analysis was conducted by using the ABI Quantitative 3.0 system to detect E1A (AD11‐F (TTGGACGGCTCCTGGAATAG), AD11‐R (TGTGACGGAAACAACCCTGACT), AD5‐F (TGATCGATCCACCCAGTGAC) and AD5‐R (ATGACAAGACCTGCAACCGT)). Results were normalized to the value of the PBS‐treated group or GAPDH.

### Cytotoxicity Test

Ninety‐six‐well plates were seeded at 3000 cells per well, and naked AD or PEI‐AD were seeded at an MOI of 40 pfu per cell starting time. Cell viability on day 3 after infection was detected by CCK‐8 assay (APExBIO).

### Virus‐to‐Cell Transfection Assay

HCT 116 cells were seeded in a 12‐well plate at 5 × 10^5^ cells per well and cultured overnight. The prepared PEI‐AD11 and naked AD11 were incubated with PBS and 1:800 titer of neutralizing antibody for 2 h, respectively. Subsequently, PEI‐AD11, naked AD11, and PEI‐AD11 pre‐incubated with neutralizing antibody, and naked AD11 pre‐incubated with neutralizing antibody were added at an initial MOI of 40 pfu per cell, respectively. After 4 h of incubation, the medium was removed, the cells were washed with PBS, and a fresh medium was added. After the cells were cultured for another 24 h, the GFP positive rate of the cells was detected and observed by flow cytometry (CytoFlex S, Beckman Coulter) and Confocal laser scanning microscopy (CLSM, Olympus IXplore SpinSR). The percentage of positive cells was scored as follows: <5% (0), 5%−25% (1), >25%−50% (2), >50%−75% (3), and >75% (4).

### In Vivo Safety Studies of Formulations

C57 mice were injected intravenously with naked AD5, PEI‐AD5, and RBC‐PEI‐AD5 once at a dose of 3 × 10^8^ pfu AD5 or equivalent PBS. 72 h after injection, the whole blood of each mouse was collected, and blood biochemistry was determined. Whole blood was centrifuged at 1500 g for 10 min at 4 °C to obtain serum, and the concentration of cytokines including IL‐6, IFN‐*γ*, and TNF‐*α* was determined by the ELISA kit according to the manufacturer's protocols. Then the animals were sacrificed, the heart was perfused, and the main organs (heart, liver, spleen, lung, and kidney) were taken out, fixed in 4% paraformaldehyde, embedded in paraffin, tissue sections, stained with H&E, photographed under a microscope, and observed for each organ organizational structure.

### In Vivo Pharmacokinetics and Biodistribution Studies

The OV formulations prepared in each group were intravenously injected into C57 mice, blood was collected from the submandibular vein at different time points (5, 30 min, 1, 2, 4, 8 h), and blood was extracted using AxyPrep Blood Genomic DNA Miniprep Kit (Axygen, China). The amount of OV in the blood was detected by qPCR at different time points. Results were shown as a percentage of the initial dose (%ID). For tissue distribution studies, 10^6^ TC‐1‐hCD46‐Luc cells were intravenously injected into the tail vein of C57BL/6J mice, and 14 days after inoculation, the prepared OV formulations of each group were intravenously injected into C57 mice. After 4 h of cardiac perfusion, the main organs of the mice (heart, liver, spleen, lung, and kidney) were taken out. DNA was collected with Fast‐pure Cell/Tissue DNA Isolation Mini Kit (Vazyme, China), then qPCR assay was performed and the native Ad11 group served as control.

### Antitumor Efficacy of EleOVt in Pulmonary Metastasis

Pulmonary metastasis was generated by intravenous injection of 10^6^ TC‐1‐hCD46‐Luc cells into C57 mice. After 3 days, the mice were randomly divided into 4 groups. RBC‐PEI‐AD11, PEI‐AD11, and naked AD11 were injected intravenously with 5 × 10^7^ pfu AD11 or the same amount of PBS every other day for a total of 6 times. Bioluminescence imaging of mice was performed using via In Vivo Imaging System (IVIS) (PerkinElmer, USA) to monitor metastatic tumor progression.

### Flow Cytometry

Lung samples were harvested after the third treatment. After the lysis of erythrocytes and live‐dead staining (Cat: 423105, Biolegend), the antibody staining process followed the manufacturer's instructions. The stained cells were measured on CytoFlex LX Flow Cytometer and the data were analyzed with FlowJo V10 software (Figures S20–S22, Supporting Information).

### Statistical Analysis

Statistical analysis was carried out using Graph Pad Prism 8. All the data were presented as mean ± standard deviation (SD). The difference between the two groups was analyzed using the Student's *t*‐test. One‐way analysis of variance (ANOVA) followed by Tukey post hoc analysis was used for multiple group comparison. The sample size for each statistical analysis and pre‐processing of data were presented in the figure legend. Differences were considered statistically significant when the *p*‐value was < 0.05.

## Conflict of Interest

The authors declare no conflict of interest.

## Author Contributions

M.L., R.Z., and H.H. contributed equally to this work. F.L., Z.P., and Z.W. performed conceptualization. F.L., D.O., and Z.P. performed the methodology. M.L., R.Z., and H.H. performed the investigation. M.L., R.Z., and H.H. performed visualization. F.L. and Z.P. performed supervision. M.L., P.L., X.Z., O.E., H.L., and H.W. performed data analysis. M.L., R.Z., and H.H. wrote the original draft. Y.H., R.X., X.Q., F.C., and Z.C. reviewed and edited the manuscript.

## Supporting information

Supporting Information

## Data Availability

The data that support the findings of this study are available from the corresponding author upon reasonable request.
