# Supplementary material for: Erythrocyte‐Leveraged Oncolytic Virotherapy (ELeOVt): Oncolytic Virus Assembly on Erythrocyte Surface to Combat Pulmonary Metastasis and Alleviate Side Effects
Source: Adv Sci (Weinh). 2023 Nov 23;11(5):2303907. doi: 10.1002/advs.202303907 (PMC10837356; doi:10.1002/advs.202303907)
Supplement: Supplementary file 1 — Supporting Information [file ADVS-11-2303907-s001.pdf]

ADVANCED SCIENCE
Open Access

Supporting Information

for *Adv. Sci.*, DOI 10.1002/advs.202303907

Erythrocyte-Leveraged Oncolytic Virotherapy (ELeOVt): Oncolytic Virus Assembly on Erythrocyte Surface to Combat Pulmonary Metastasis and Alleviate Side Effects

Mingyang Liu, Ruizhe Zhang, Hanwei Huang, Pengfei Liu, Xu Zhao, Hu Wu, Ying He, Ruizhe Xu, Xifeng Qin, Zhenguo Cheng, Hongyu Liu, Onder Ergonul, Füsun Can, Defang Ouyang, Zhenning Wang, Zhiqing Pang* and Funan Liu**

Supplementary Materials for

Erythrocyte-leveraged oncolytic virotherapy (ELeOVt): Oncolytic Virus assembly on erythrocyte surface to combat pulmonary metastasis and alleviate side effects

Mingyang Liu[#], Ruizhe Zhang[#], Hanwei Huang[#], Pengfei Liu, Xu Zhao, Hu Wu, Ying He, Ruizhe Xu, Xifeng Qin, Zhenguo Cheng, Hongyu Liu, Onder Ergonul, Füsun Can, Defang Ouyang, Zhenning Wang[*], Zhiqing Pang[*], Funan Liu[*]

*Corresponding author: Funan Liu Email: fnliu@cmu.edu.cn

Zhiqing Pang Email: zqpang@fudan.edu.cn

Zhenning Wang Email: znwang@cmu.edu.cn

Supplementary Text

Experimental Sections

Blood collection and processing. Mouse whole blood was collected through the mouse orbit using capillary tubes pre-soaked with heparin sodium PBS solution and stored in heparin sodium blood collection tubes until use. Whole blood was centrifuged at 400g for 10 min at 4°C to remove the serum and the buffy coat layers from the erythrocyte compartment. The isolated erythrocytes were further washed three times with cold PBS and centrifuged at 500g for 5 min at 4°C before their final resuspension at a concentration of 10% hematocrit in PBS (erythrocyte stock solution). Freshly processed erythrocytes were used for each experiment in this study.

***In vitro* serum stability and shear studies.** For serum stability studies, hitchhiked mouse and human erythrocytes were incubated in 1 ml of fetal bovine serum (FBS) or human serum (from IPHASE, China) on a longitudinal axis (Rolling Incubator QB-328, Kylin-Bell Lab Instruments Co., Ltd., China) at 15 rpm at 37°C. These conditions simulate low shear physiological environment. After incubation for 30 min, the cells were pelleted by centrifugation at 500g for 5 min and resuspended to 10% (v/v) in 1× PBS. For shear studies, hitchhiked mouse and human erythrocytes were incubated in 10 ml of FBS or human serum. A rotatory shear (6 Pa) was applied to erythrocytes in serum using a cylindrical coquette viscometer (1 mm gap, AR-G2 rheometer, TA instruments, DE, USA) for 30 min. The samples were maintained at 37°C during the application of shear using a water bath. These conditions simulate lung-corresponding high shear physiological environment. After 30 min, the cells were pelleted by centrifugation at 500g for 5 min and resuspended to 10% (v/v) in 1× PBS. After DNA extraction from the preparations using AxyPrep™ Blood Genomic DNA Miniprep Kit (Axygen, China), qPCR was used to determine the count of AD11 remaining in preparations subjected to a series of shear stress studies. The formulation prior to the shear stress study was used as a control.

Cell endocytosis assay. Cells endocytosis assay was performed as described previously[1]. Briefly, Raw 264.7 cells were seeded at 10^6 cells per well into a 12-well plate. Naked AD, PEI-AD and RBC-PEI-AD were added at a starting MOI of 20 pfu/cell. After 4 h of incubation, cells were washed with ice-cold acetate buffer and PBS to wash away adsorbed AD on the cell surface. Then DNA was collected with Fast-pure® Cell/Tissue DNA Isolation Mini Kit (Vazyme, China).

The amount of OV endocytosed was determined by qPCR. The uptake of TC-1 for different formulations was also measured using the same method.

Immunohistochemistry. At the end of pharmacodynamics study, immunohistochemistry (IHC) staining of lung slices was performed as previously described[2]. The sections from lungs were deparaffinized, rehydrated, and heated for antigen retrieval and to block endogenous peroxidases. After blocking in 10% normal goat serum, sections were stained with hematoxylin and eosin.

Immunofluorescence imaging. At the end of pharmacodynamics study, immunofluorescence staining of T cells was performed on 4-μm paraffin sections of lungs as previously reported[2]. Briefly, the section was successively stained with primary antibodies against $CD4^+$ or $CD8^+$ cells (1:400, Abcam, USA), the fluorescence-labeled secondary goat anti-rabbit antibodies (Yeasen, China) and then subjected to a fluorescent digital biopsy scanner.

[1] W. Lu, Y. Zhang, Y.-Z. Tan, K.-L. Hu, X.-G. Jiang, S.-K. Fu, *J Control Release* **2005**, 107, 428.
[2] F. Liu, Z. Cheng, X. Li, Y. Li, H. Zhang, J. Li, F. Liu, H. Xu, F. Li, *Mol Ther Nucleic Acids* **2017**, 8, 370.

Table. S1. Antibody list.

**Antibody**	**Clone**	**Company**	**Catalog No.**
**CD3**	145-2C11	Biolegend	100305
**CD4**	RM4-5	Biolegend	100527
**CD8**	53-6.7	Biolegend	100711
**CD45**	30-F11	Biolegend	103131
**IFN-γ**	XMG1.2	Biolegend	505807
**MHC II**	M5/114.15.2	Biolegend	107605
**CD11c**	N418	Biolegend	117317
**CD80**	16-10A1	Biolegend	104713
**CD86**	GL-1	Biolegend	105011
**CD16/32 TruStain FcX**	S17011E	Biolegend	156603

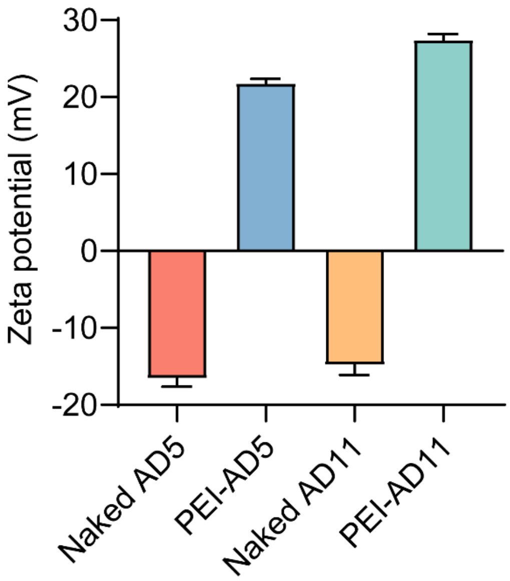

Fig. S1.

Zeta potential of naked AD5, PEI-AD5, naked AD11, and PEI-AD11, $n = 3$. Data are displayed as mean ± s.d..

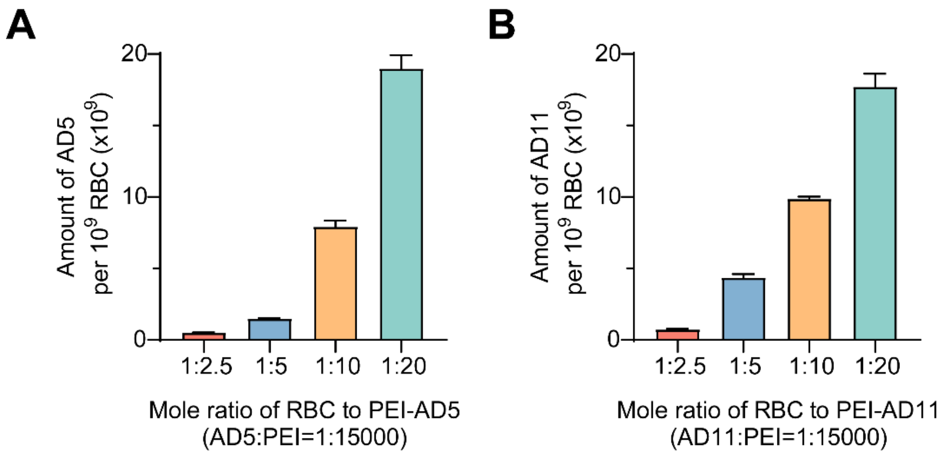

Fig. S2.

Amount of (A) AD5 and (B) AD11 on mouse erythrocytes at different mole ratios of erythrocytes to PEI-AD, $n = 3$. Data are displayed as mean ± s.d..

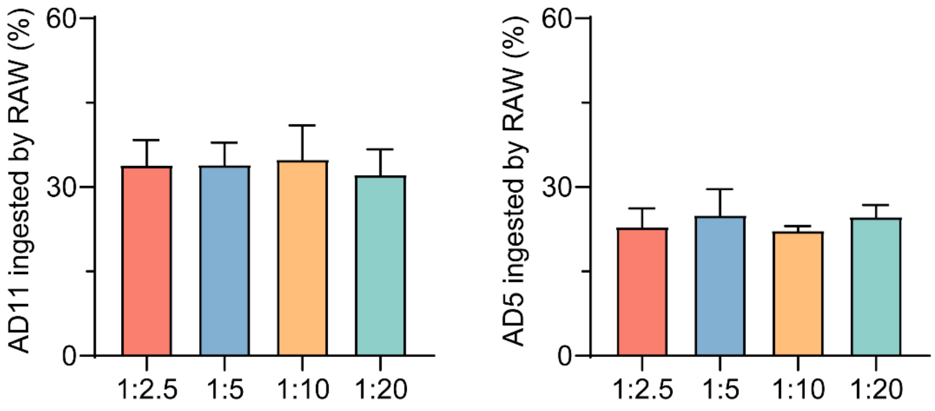

Fig. S3.

The relative percentage of RBC-PEI-AD11 or RBC-PEI-AD5 endocytosed by macrophages at different loading mole ratios of erythrocytes to PEI-AD11 or PEI-AD5, n = 3. Data are displayed as mean ± s.d.. The data are presented as a relative percentage compared to the naked AD11 or AD5 group.

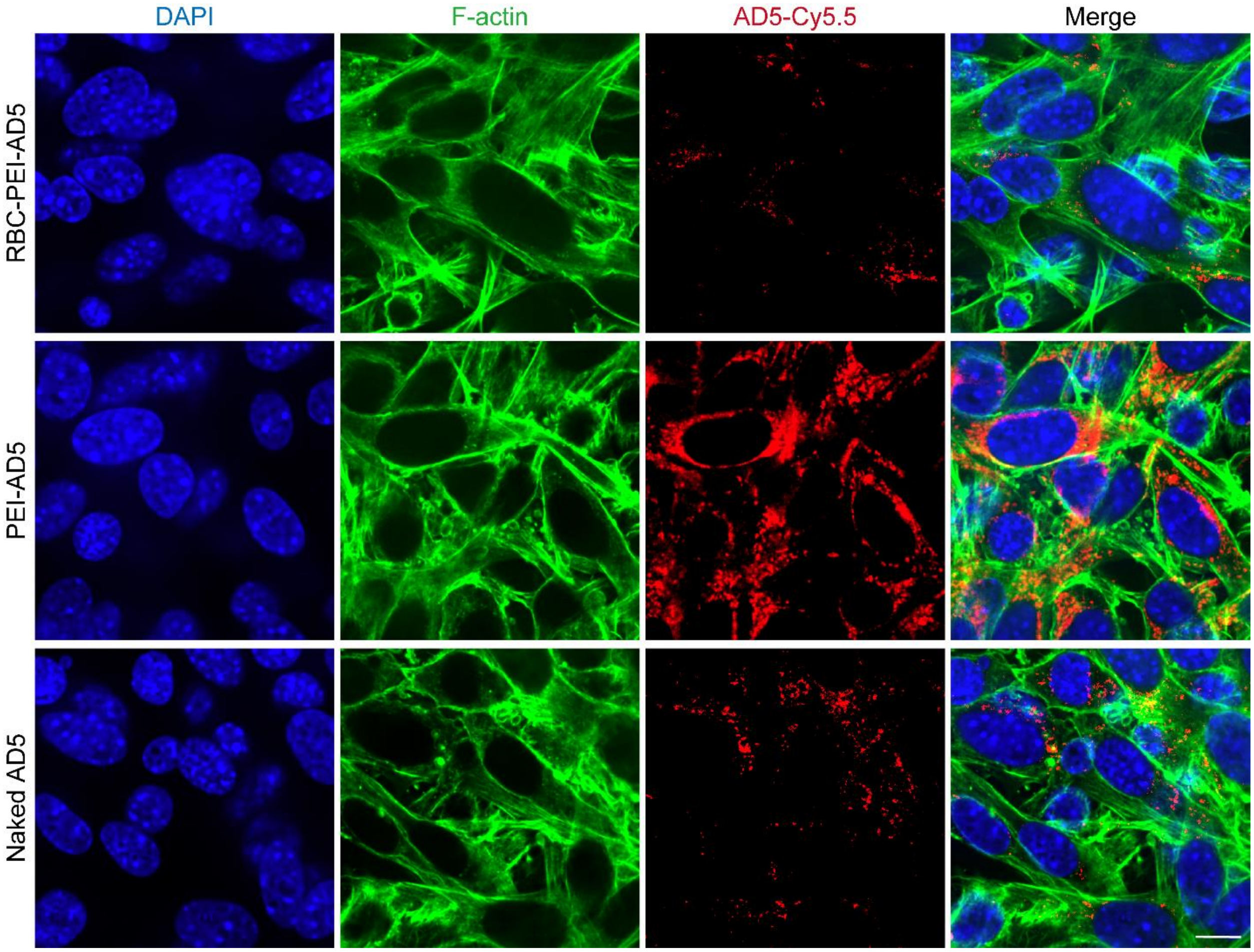

Fig. S4.

CLSM images of Cy5.5-labeled RBC-PEI-AD5, PEI-AD5, and naked AD5 endocytosed by TC-1, scale bar = 10 μm.

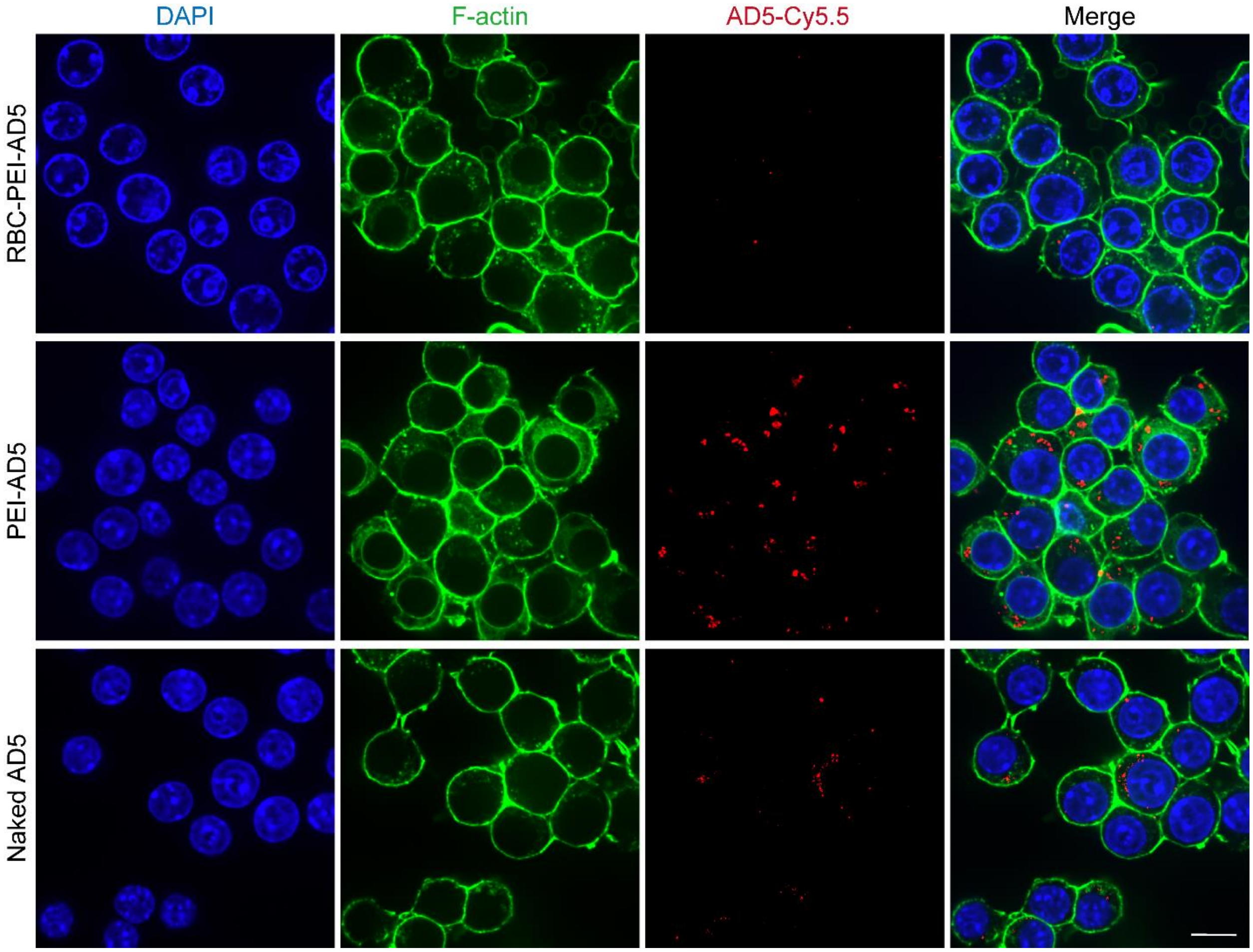

Fig. S5.

CLSM images of Cy5.5-labeled RBC-PEI-AD5, PEI-AD5, and naked AD5 endocytosed by macrophages, scale bar = 10 μm.

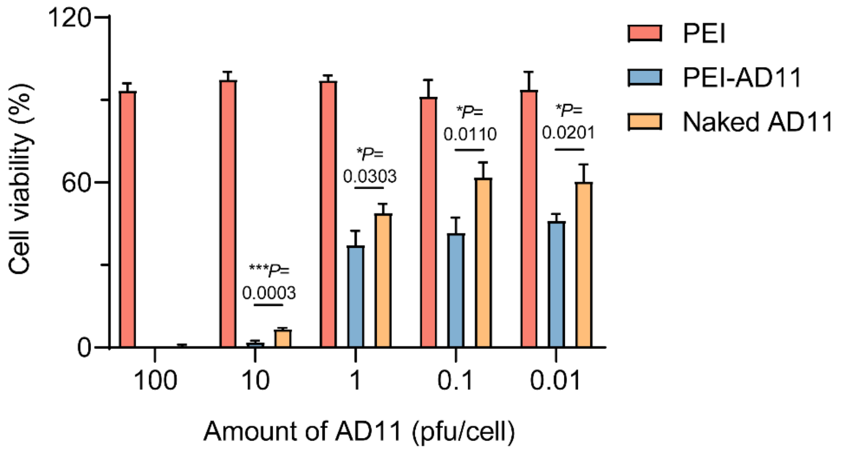

Fig. S6.

In vitro cytotoxicity of PEI, PEI-AD11 and naked AD11 on TC-1 cells after incubation for 72 h, n = 3. Data are displayed as mean ± s.d.. Statistical significance was analyzed by a two-tailed Student's t-test. P-value: *$P < 0.05$, ***$P < 0.001$.

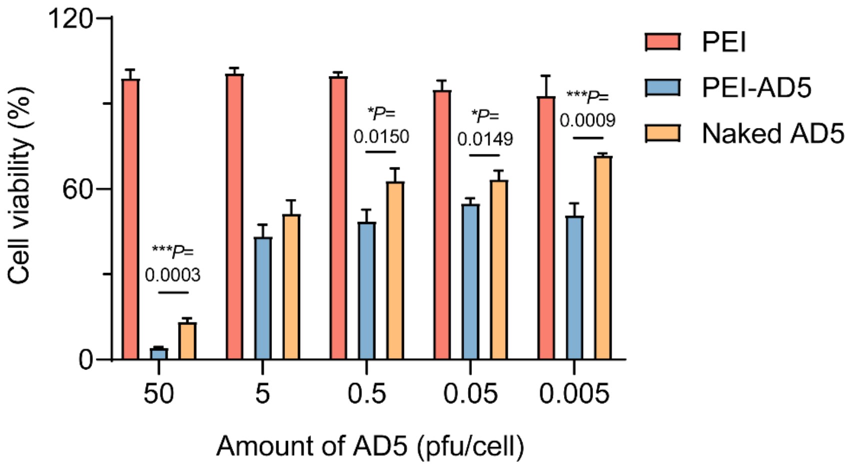

Fig. S7.

In vitro cytotoxicity of PEI, PEI-AD5 and naked AD5 on HCT 116 cells after incubation for 72 h, $n = 3$. Data are displayed as mean ± s.d.. Statistical significance was analyzed by a two-tailed Student's t-test. P-value: *$P < 0.05$, ***$P < 0.001$.

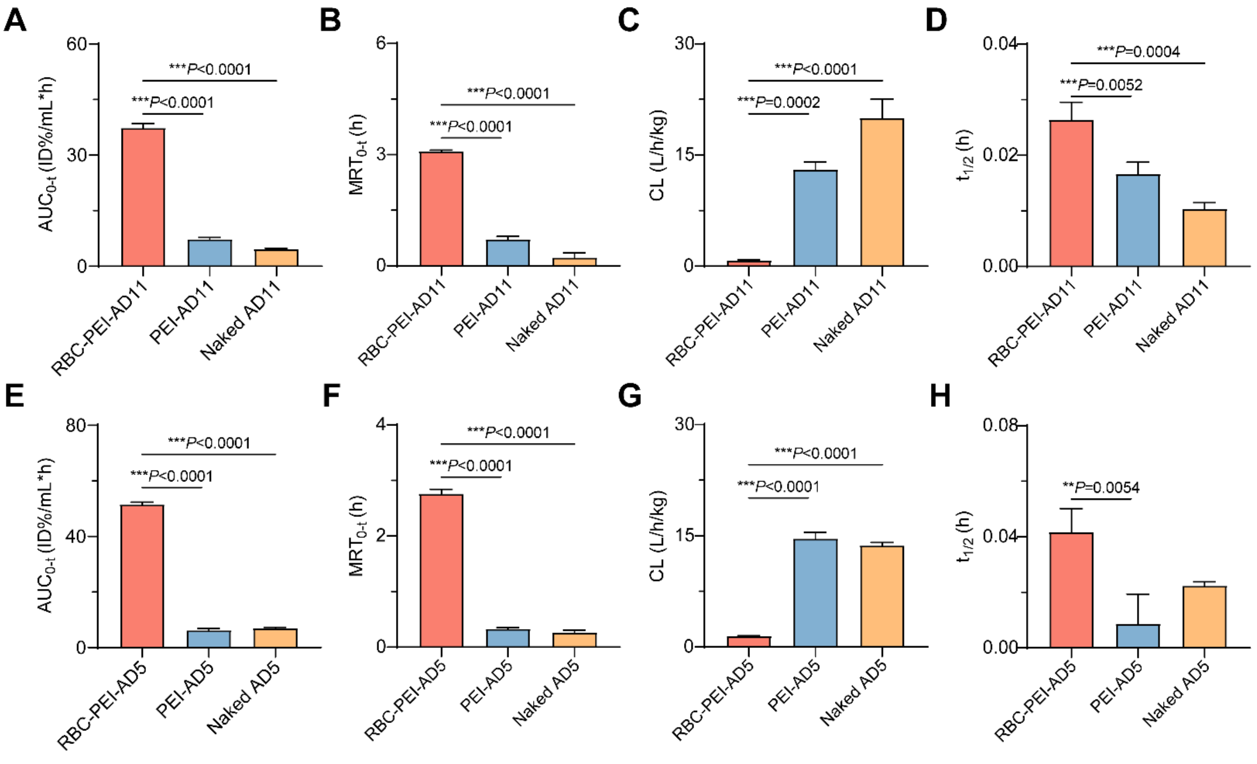

Fig. S8.

(A-D) The (A) $AUC_{0\text{-}t}$, (B) $MRT_{0\text{-}t}$, (C) CL, and (D) $t_{1/2}$ for the RBC-PEI-AD11, PEI-AD11 and naked AD11 after intravenous injection, $n = 3$. (E-H) The (E) $AUC_{0\text{-}t}$, (F) $MRT_{0\text{-}t}$, (G) CL, and (H) $t_{1/2}$ for the RBC-PEI-AD5, PEI-AD5 and naked AD5 after intravenous injection, $n = 3$. Data are displayed as mean ± s.d.. Statistical significance was analyzed by one-way ANOVA with a Tukey *post hoc* test. *P*-value: $^{**}P < 0.01$, $^{***}P < 0.001$.

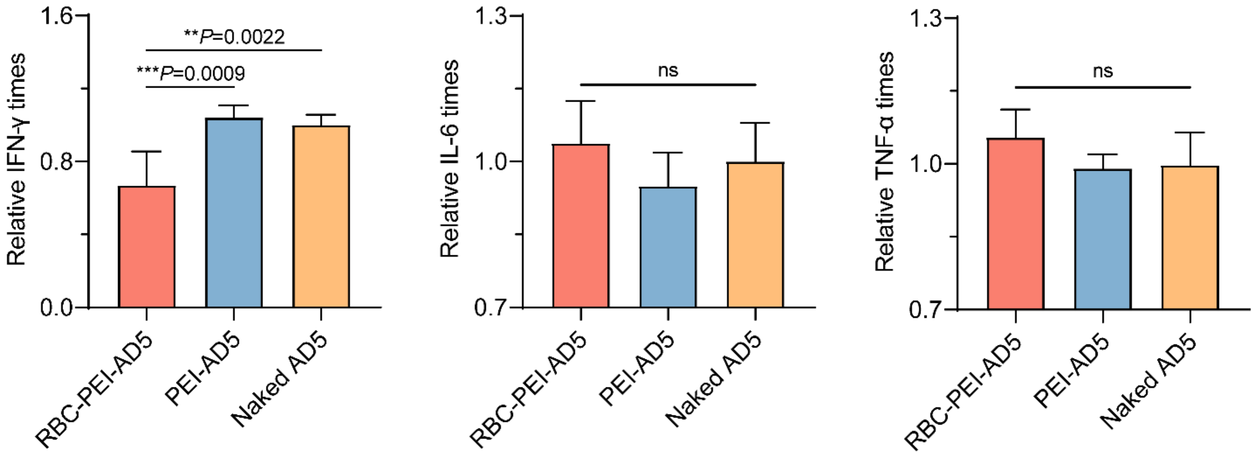

Fig. S9.

The concentrations of IFN-γ, IL-6, and TNF-α in the peripheral blood at 72h after intravenous administration of different AD5 formulations, n = 5. Data are displayed as mean ± s.d. and are presented as relative times compared to the naked AD5 group. Statistical significance was analyzed by one-way ANOVA with a Tukey *post hoc* test, ns means no significant difference. P-value: $^{**}P < 0.01$, $^{***}P < 0.001$.

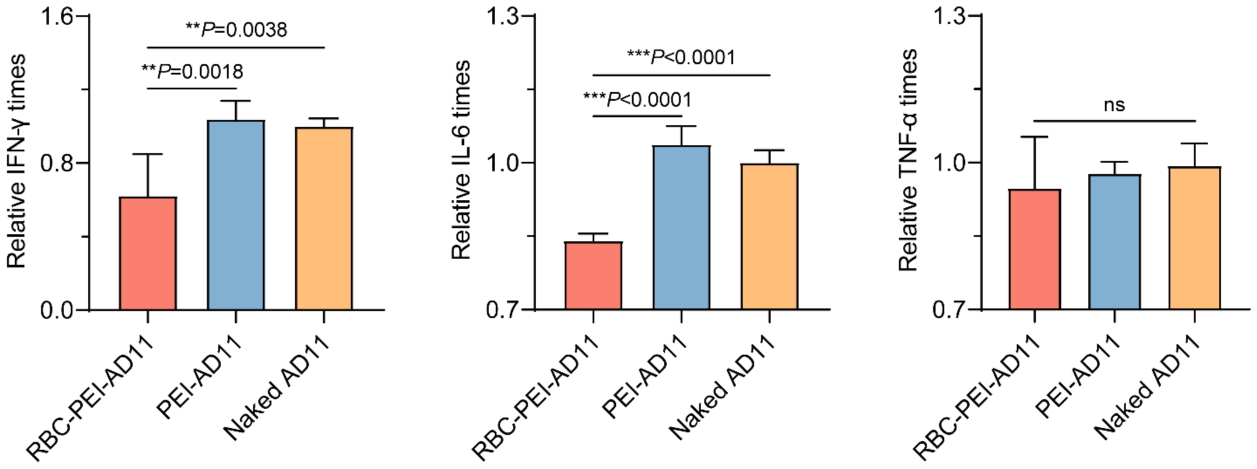

Fig. S10.

The concentrations of IFN-γ, IL-6, and TNF-α in the peripheral blood at 72h after intravenous administration of different AD11 formulations, n = 5. Data are displayed as mean ± s.d. and are presented as relative times compared to the naked AD11 group. Statistical significance was analyzed by one-way ANOVA with a Tukey *post hoc* test, ns means no significant difference. P-value: $^{**}P < 0.01$, $^{***}P < 0.001$.

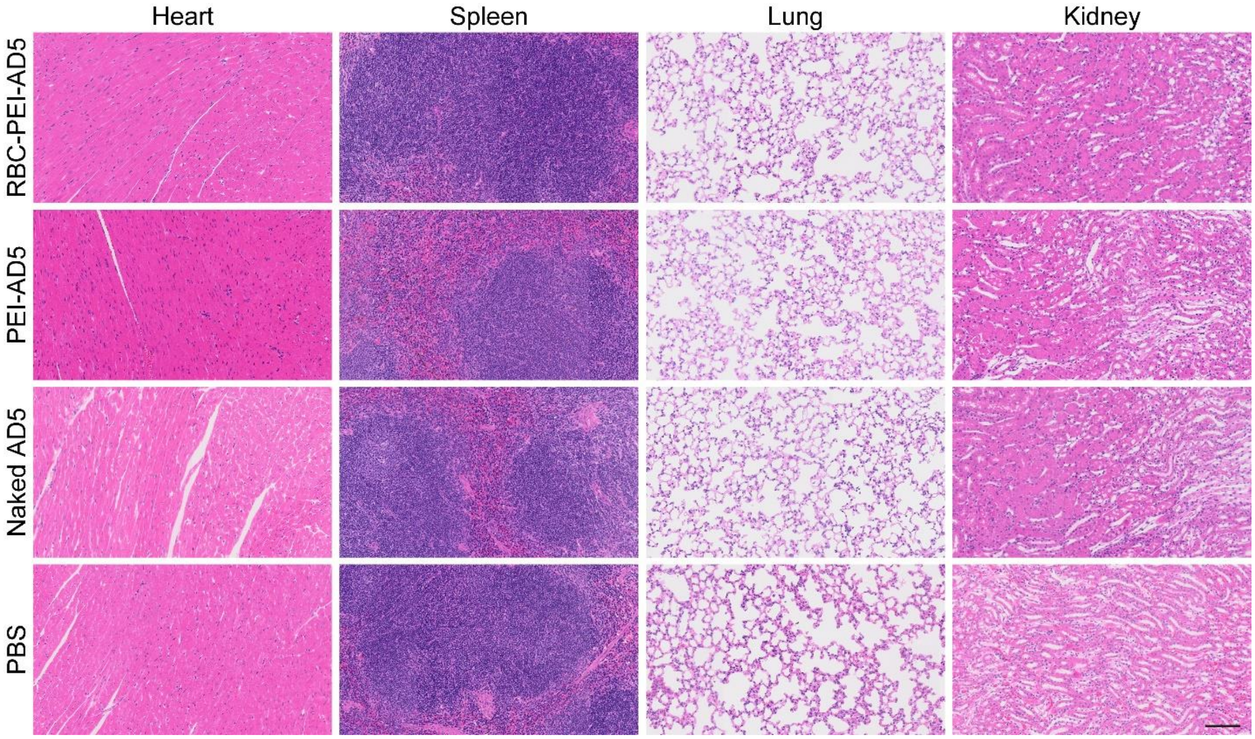

Fig. S11.

The histopathological analysis of major organs including the heart, spleen, lung, and kidneys by H&E staining at 72 h after intravenous administration of a high-dose of different AD5 formulations, scale bar = 100 μm.

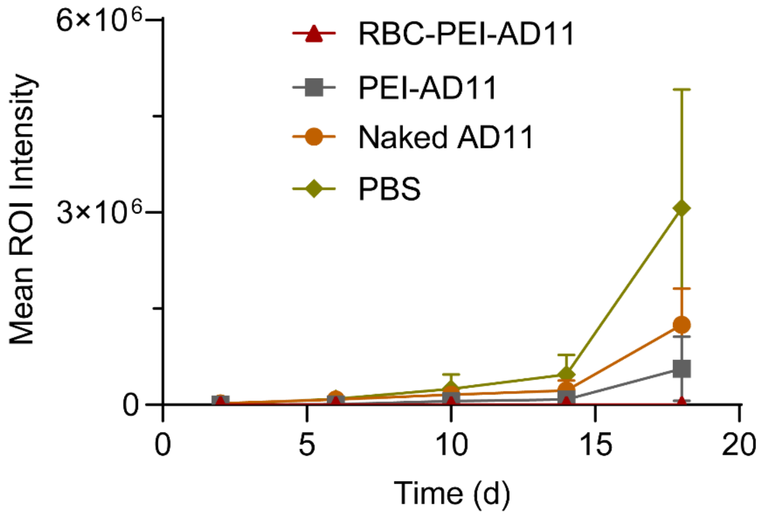

Fig. S12.

Quantification of bioluminescence intensity of mice in different groups, $n = 5$. Data are displayed as mean ± s.d..

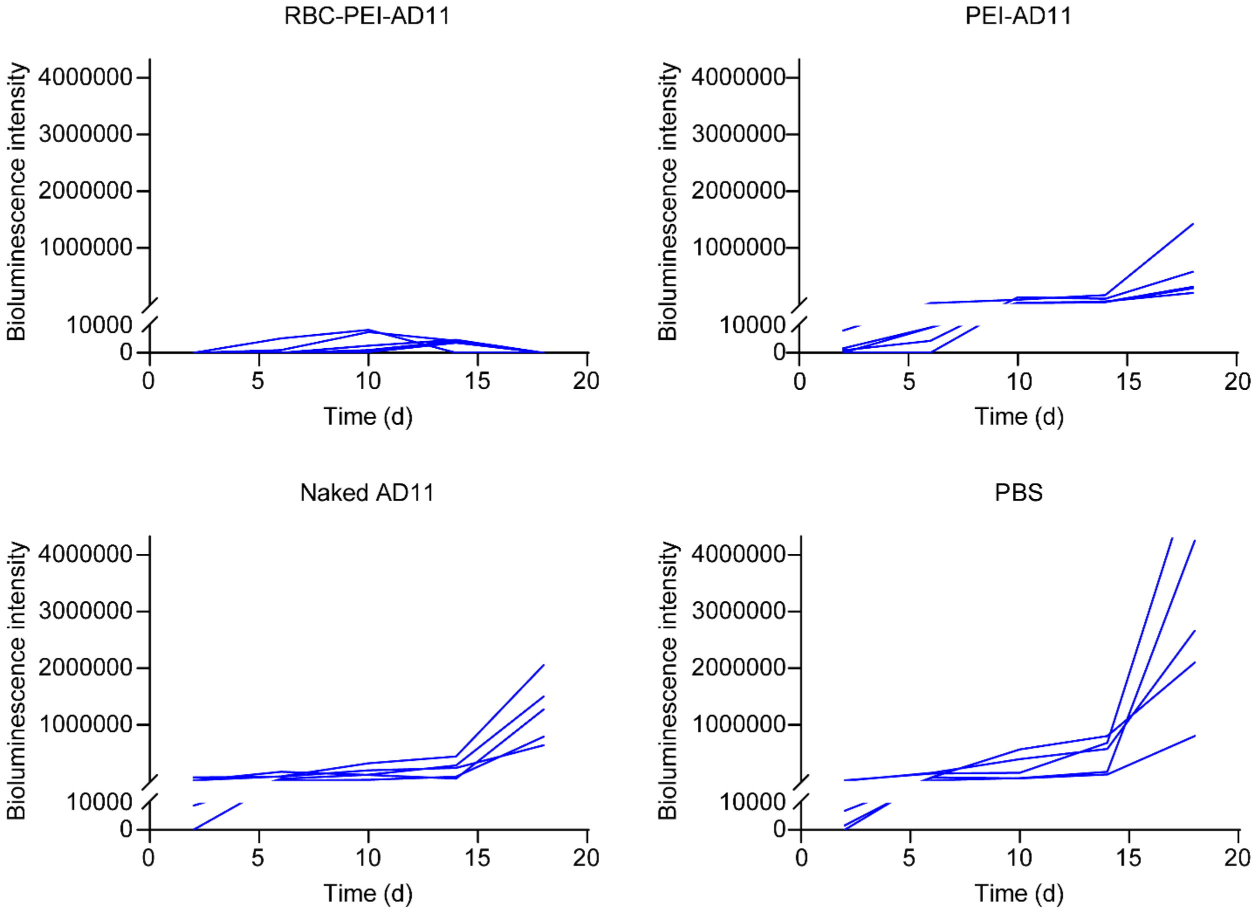

Fig. S13.
Quantification of bioluminescence intensity of each mouse in different groups, $n = 5$.

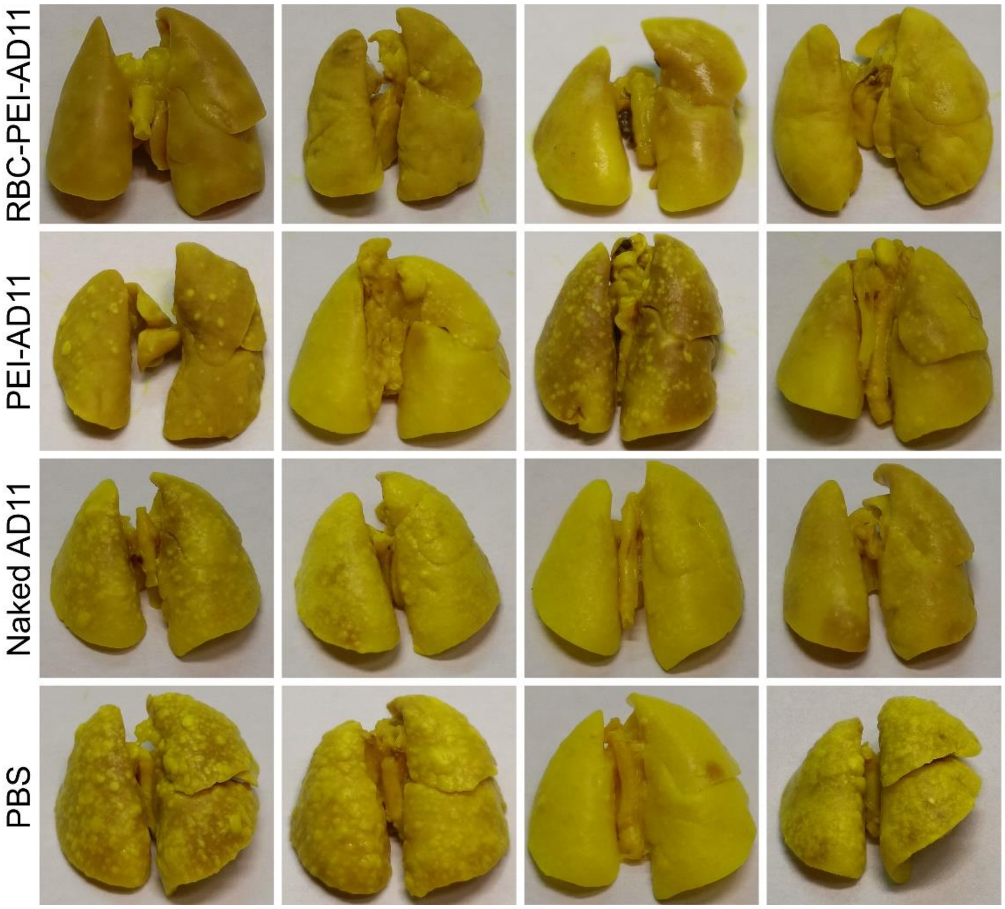

Fig. S14.

The photographs of lungs other than those shown in the main text.

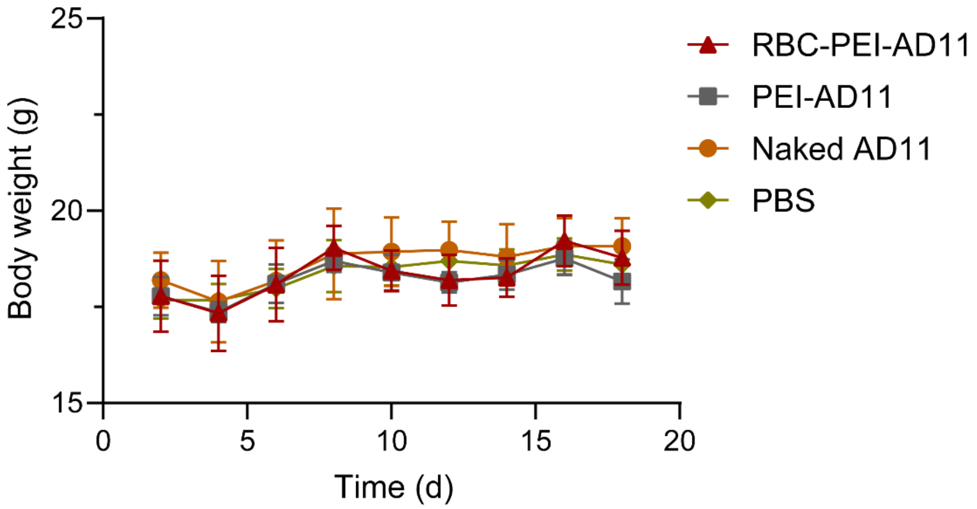

Fig. S15.

Body weight change of mice during the treatment period, n = 5. Data are displayed as mean ± s.d..

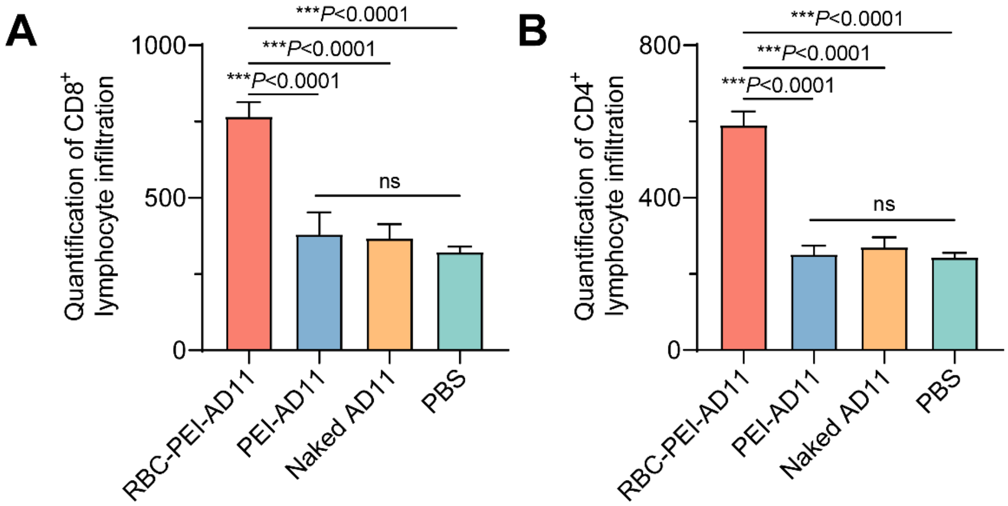

Fig. S16.

(A, B) The number of (A) $CD8^+$ and (B) $CD4^+$ T cells in immunofluorescence staining images of lungs after different treatments, $n = 3$. Data are displayed as mean ± s.d.. Statistical significance was analyzed by one-way ANOVA with a Tukey *post hoc* test; ns means no significant difference. P-value: $***P < 0.001$.

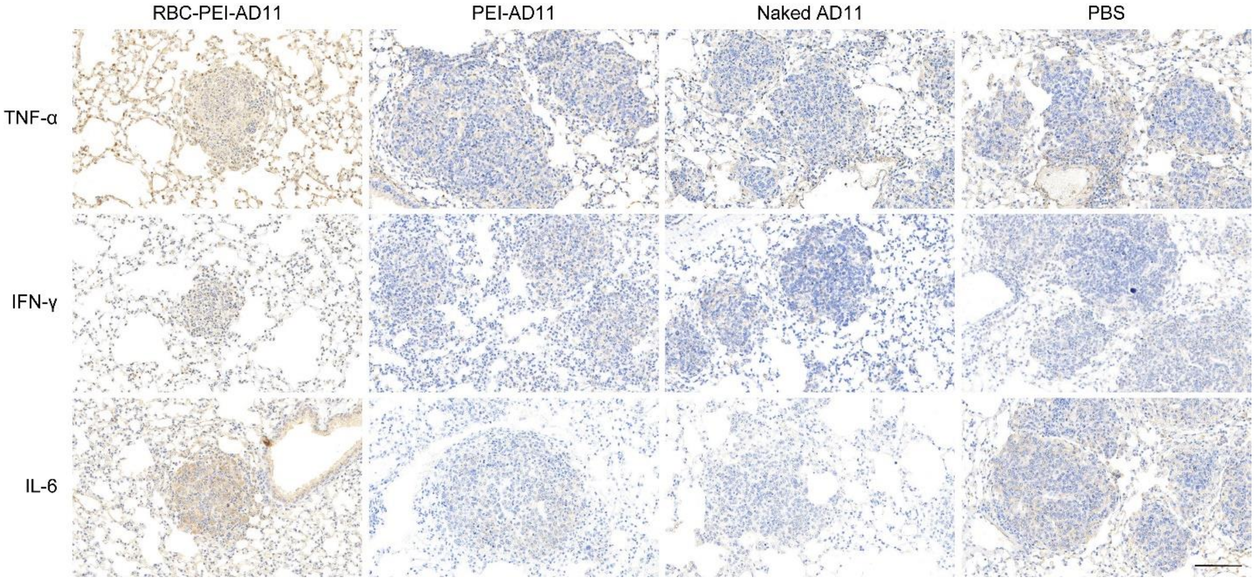

Fig. S17.

The representative immunohistochemical staining images of lung metastatic tumors after different treatments. Scale bar = 100 μm.

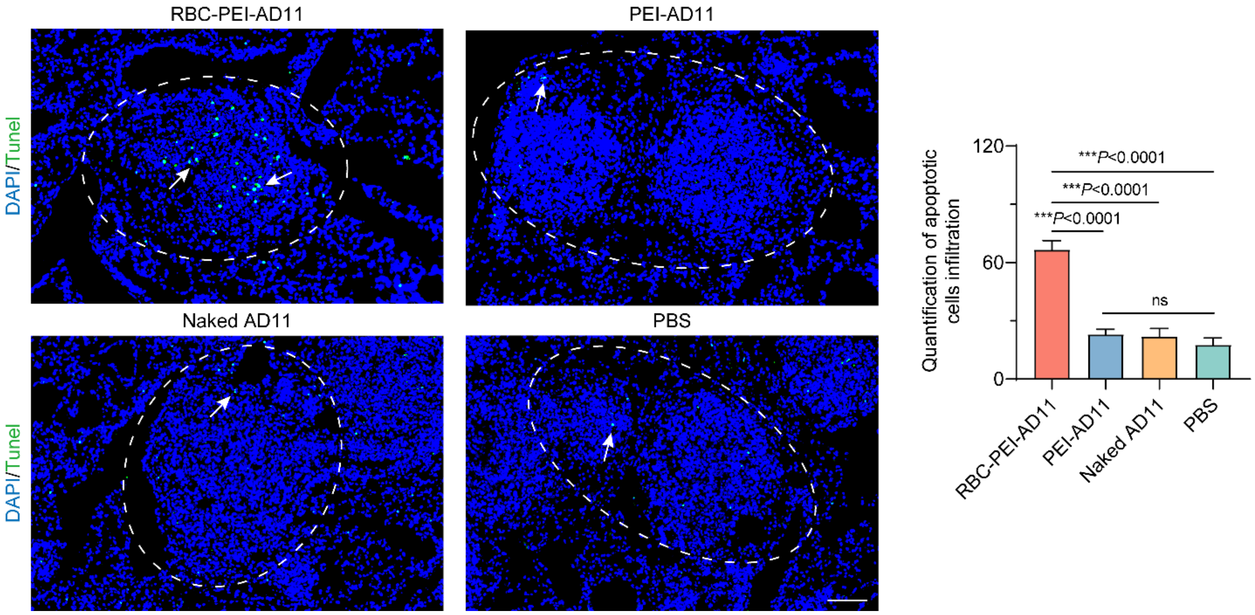

Fig. S18.

Representative images of apoptotic cells and the number of apoptotic cells in lung metastatic nodes after different treatments. The lung metastatic node was indicated with the dotted oval and apoptotic cell was marked by the white arrow, $n = 3$, scale bar = 100 μm. Data are displayed as mean ± s.d.. Statistical significance was analyzed by one-way ANOVA with a Tukey *post hoc* test; ns means no significant difference. *P*-value: $***P < 0.001$.

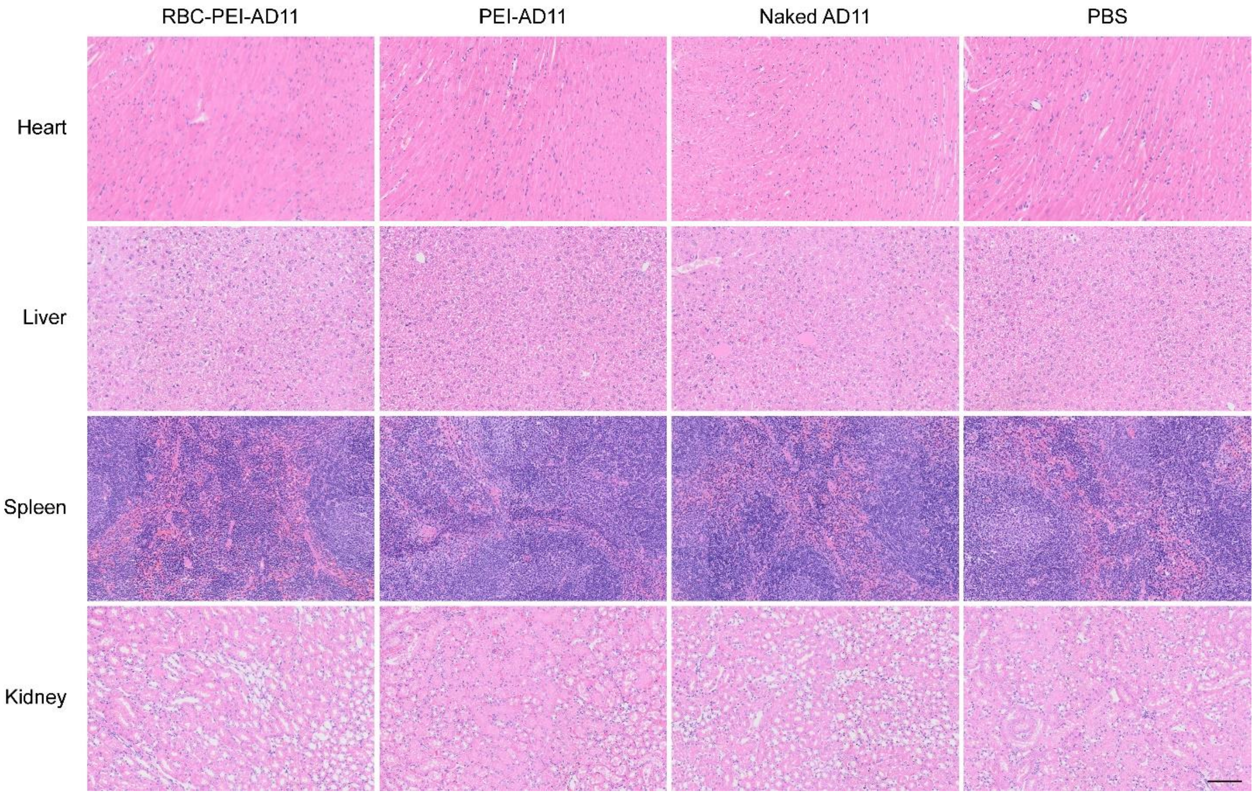

Fig. S19.

Histopathological analysis of major organs including the heart, liver, spleen, and kidneys in different treatment groups by H&E staining at day 18 after lung metastases were implanted through the caudal vein, scale bar = 100 μm.

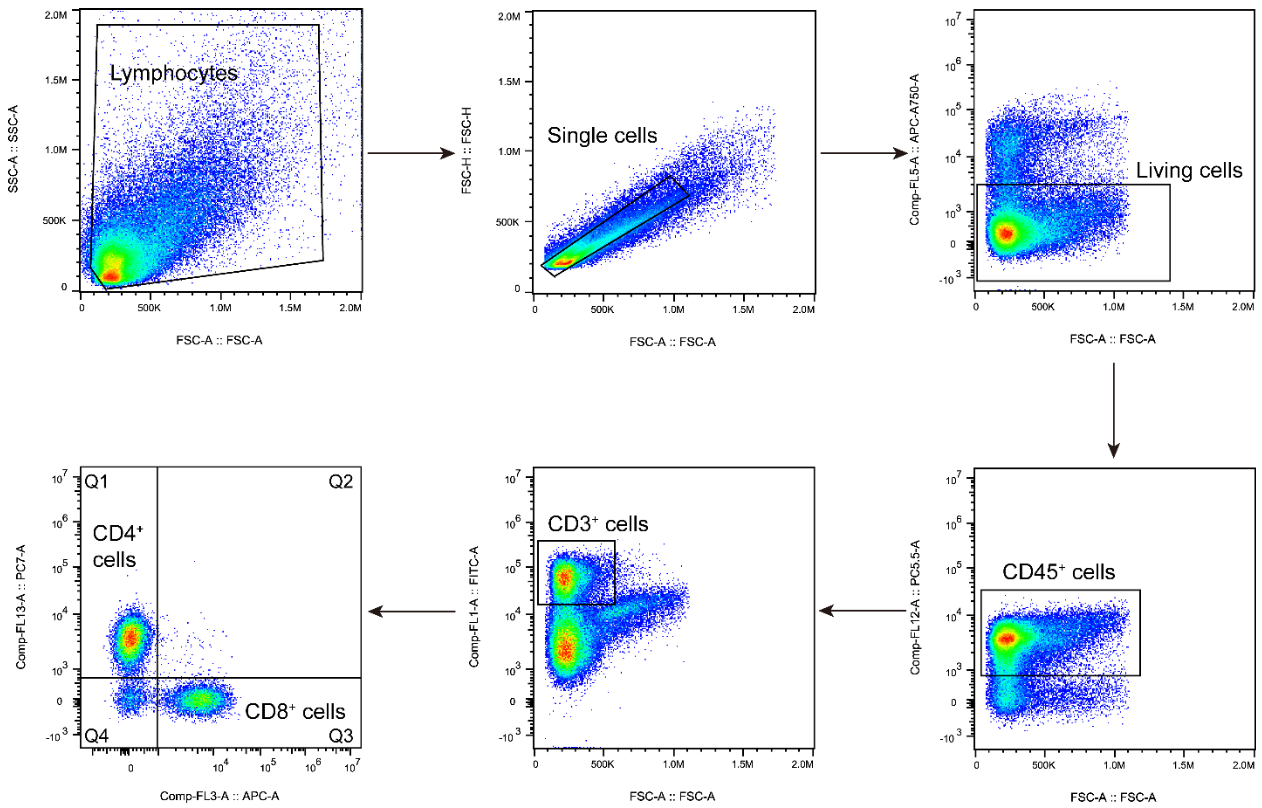

Fig. S20.

Gating strategy for the analysis of CD4$^+$ T cells and CD8$^+$ T cells in lungs.

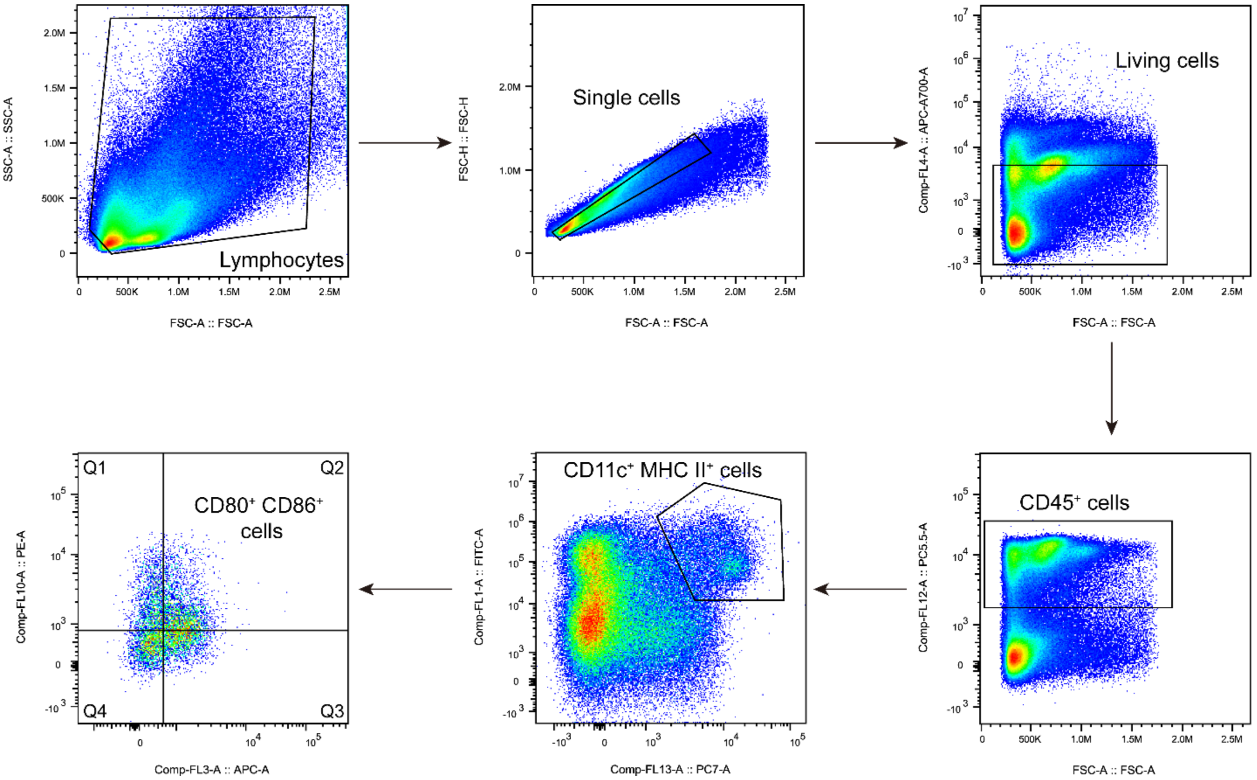

Fig. S21.

Gating strategy for the analysis of DCs in lungs.

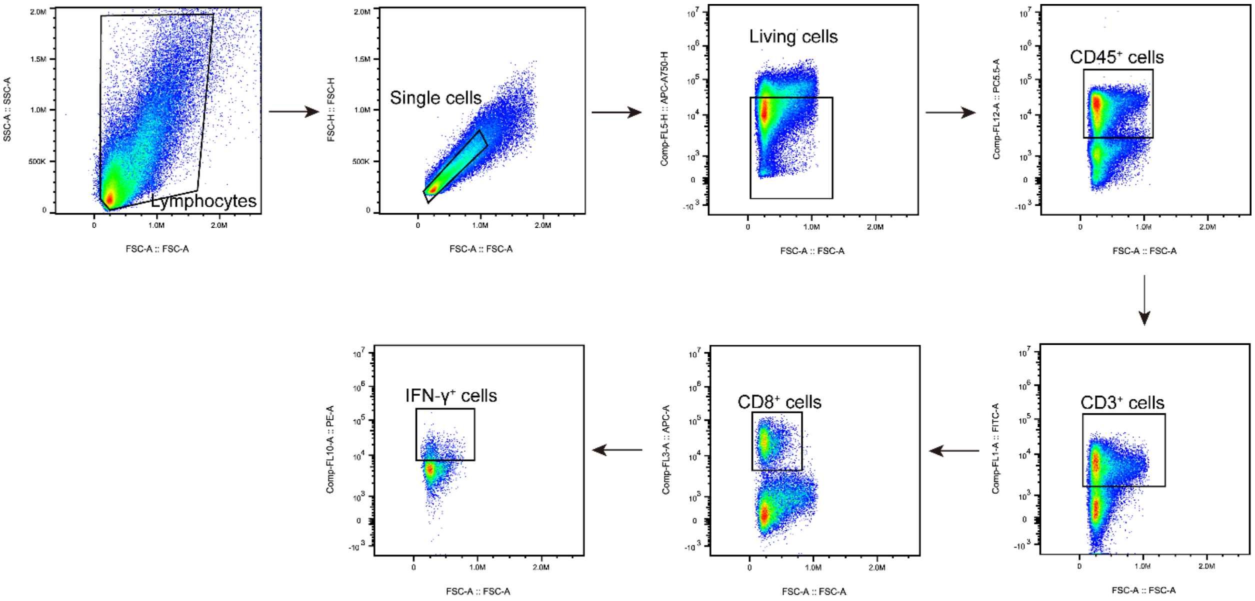

Fig. S22.

Gating strategy for the analysis of IFN-γ^+ CD8$^+$ T cells in lungs.
